# *In silico* assessment of pharmacotherapy for carbon monoxide induced arrhythmias in healthy and failing human hearts

**DOI:** 10.3389/fphys.2022.1018299

**Published:** 2022-11-16

**Authors:** Huasen Jiang, Shugang Zhang, Weigang Lu, Fei Yang, Xiangpeng Bi, Wenjian Ma, Zhiqiang Wei

**Affiliations:** ^1^ College of Computer Science and Technology, Ocean University of China, Qingdao, China; ^2^ Department of Educational Technology, Ocean University of China, Qingdao, China; ^3^ School of Mechanical, Electrical, and Information Engineering, Shandong University, Weihai, China

**Keywords:** carbon monoxide, pharmacotherapy, simulation, arrhythmia, air pollution

## Abstract

**Background:** Carbon monoxide (CO) is gaining increased attention in air pollution-induced arrhythmias. The severe cardiotoxic consequences of CO urgently require effective pharmacotherapy to treat it. However, existing evidence demonstrates that CO can induce arrhythmias by directly affecting multiple ion channels, which is a pathway distinct from heart ischemia and has received less concern in clinical treatment.

**Objective:** To evaluate the efficacy of some common clinical antiarrhythmic drugs for CO-induced arrhythmias, and to propose a potential pharmacotherapy for CO-induced arrhythmias through the virtual pathological cell and tissue models.

**Methods:** Two pathological models describing CO effects on healthy and failing hearts were constructed as control baseline models. After this, we first assessed the efficacy of some common antiarrhythmic drugs like ranolazine, amiodarone, nifedipine, etc., by incorporating their ion channel-level effects into the cell model. Cellular biomarkers like action potential duration and tissue-level biomarkers such as the QT interval from pseudo-ECGs were obtained to assess the drug efficacy. In addition, we also evaluated multiple specific *I*_Kr_ activators in a similar way to multi-channel blocking drugs, as the *I*_Kr_ activator showed great potency in dealing with CO-induced pathological changes.

**Results:** Simulation results showed that the tested seven antiarrhythmic drugs failed to rescue the heart from CO-induced arrhythmias in terms of the action potential and the ECG manifestation. Some of them even worsened the condition of arrhythmogenesis. In contrast, *I*_Kr_ activators like HW-0168 effectively alleviated the proarrhythmic effects of CO.

**Conclusion:** Current antiarrhythmic drugs including the ranolazine suggested in previous studies did not achieve therapeutic effects for the cardiotoxicity of CO, and we showed that the specific *I*_Kr_ activator is a promising pharmacotherapy for the treatment of CO-induced arrhythmias.

## 1 Introduction

Carbon monoxide (CO) is one of the major gaseous pollutants in traffic pollution. Epidemiological studies have substantiated the association of urban air pollution with cardiovascular events, among which CO is considered as a critical contributor (Hoek et al., 2002; Hoffmann et al., 2007; Allen et al., 2009; Bell et al., 2009). The traditional theory of CO poisoning attributes CO-induced arrhythmias to tissue hypoxia, a condition that arises from the high-affinity binding of CO to hemoglobin, which may predispose to arrhythmias (Hantson, 2019). However, accumulating evidences have demonstrated that CO can also impair cardiac electrophysiology by exerting direct effects on multiple ion channels. For sodium channels, Dallas et al. demonstrated that CO could enhance the late Na^+^ current (*I*_NaL_) by increasing the production of NO and the subsequent nitrosylation of the Na_V_1.5 channel protein (Dallas et al., 2012). In addition, CO could inhibit the *I*_Na_ and the process was dependent on the NO formation and channel redox states (Elies et al., 2014). For calcium channels, Scragg et al. found that CO inhibited L-type Ca^2+^ channels (*I*_CaL_) *via* redox modulation of key cysteine residues by mitochondrial reactive oxygen species (Scragg et al., 2008). Finally, for potassium channels, CO inhibited inward rectifier K^+^ current (*I*_K1_) by modulating the interaction between Kir2.0 channels and phosphatidylinositol (4, 5)-diphosphate (Liang et al., 2014), and inhibited the rapid delayed rectifier K^+^ current (*I*_Kr_) by promoting the production of peroxynitrite (ONOO^−^) (Al-Owais et al., 2017). These remodeling effects together contributed to a prolonged QT interval and predisposed to severe ventricular arrhythmias like Torsades de Pointes (TdP) (Jiang et al., 2022). Such arrhythmogenic influences may get even worse in susceptible populations like heart failure (HF) patients. This is because the repolarization reserve has been reduced in failing hearts, and the further depression of *I*_Kr_ by CO can easily lead to early-afterdepolarization (EAD) activities in cardiomyocytes and ectopic beats at the organ level, which act as triggers for reentry arrhythmias (Al-Owais et al., 2021).

The serious consequence of CO cardiotoxicity has raised concerns on finding an effective pharmacotherapy for it. In this regard, potential drugs have been raised to deal with the proarrhythmic effects of CO. For instance, the antianginal drug ranolazine was suggested by Dallas et al. for its significant therapeutic effects on CO-induced arrhythmias (Dallas et al., 2012). *In vivo* experiments showed that ranolazine corrected QT variability and arrhythmias induced by CO, and further cellular investigations reported that ranolazine abolished CO-induced early after-depolarizations (EADs) in rat myocytes *via* the inhibition of *I*_NaL_. This study highlighted a potential pharmacological strategy for the treatment of CO-induced arrhythmias; however, the efficacy of ranolazine was evaluated in rats, and the significant discrepancy between rats and human action potentials may limit their conclusions. Despite that ranolazine can inhibit *I*_NaL_ and correct CO-induced arrhythmias in rat ventricular myocytes, the drug is also known to block *I*_Kr_ (IC_50_ 12 μM) (Rajamani et al., 2008) in an overlapped range with *I*_NaL_ (IC_50_ 5–21 μM) (Moreno et al., 2013). Therefore, considering the complicated multi-channel blocking effect of ranolazine, whether it still exerts antiarrhythmic effects in the human ventricle needs to be re-assessed. In addition to ranolazine, our previous simulation study on CO exposure showed that the inhibition of *I*_Kr_ by CO is the main factor responsible for the substantial prolongation of the QT interval in patients (Jiang et al., 2022). Therefore, specific *I*_Kr_ activators such as HW-0168 (Dong et al., 2019) might benefit the treatment of CO-induced arrhythmias.

In this study, we conducted an *in silico* assessment of pharmacotherapy for the treatment of CO-induced ventricular arrhythmias in healthy and failing hearts. First, human myocardial cell and tissue models with the effects of CO incorporated were constructed on healthy and heart failure conditions, respectively, to act as baseline pharmacological models for the screening of drugs. Next, we evaluated several of the clinically available antiarrhythmic drugs described above by incorporating their experimentally-measured dose-dependent effects on various ion channels. The class IV antiarrhythmic drugs (i.e., calcium channel blockers including verapamil, nifedipine, and bepridil) were mainly focused on due to their ability of attenuating depolarization forces. We also tested three other multi-channel drugs for a wide coverage of the antiarrhythmic drug classification. These drugs are namely quinidine (class I), amiodarone (class III), and vanoxerine (class III). Noted that, like the case of ranolazine, all these six drugs are multi-channel blockers and can block some critical channels concurrently. Action potentials and pseudo-ECGs after the application of drugs were simulated and used as the criteria for drug efficacy. In addition, due to the critical role of *I*_Kr_ in mediating CO-induced arrhythmogenesis, we also evaluated multiple *I*_Kr_ activators for potential pharmacotherapy. Comprehensive Simulations were conducted on cell populations, 1D transmural strands, and 2D ventricular slice models to verify the robustness of the reported findings.

## 2 Methods

### 2.1 Modeling action potentials of human ventricular myocytes

The O'Hara-Rudy dynamics (ORd) model (O’Hara et al., 2011) was utilized to simulate the electrophysiology of human ventricular myocytes in this study. The ORd model is a comprehensive human cell model that was created using human experimental data. To overcome its unphysiologically slow conduction velocity (Elshrif and Cherry, 2014), the original *I*_Na_ in the ORd model was substituted with that in the Tusscher et al. biophysically detailed model (TNNP06 model) (Ten Tusscher and Panfilov, 2006).

A conventional Hodgkin-Huxley model of a cardiac cell was implemented at the cellular level, with the model equation being:

$$\frac{\partial V_{m}}{\partial t}=-\frac{\left(I_{ion}+I_{stim}\right)}{C_{m}}$$ (1)

where *V*_*m*_ is the membrane potential, *I*_ion_ is the sum of all transmembrane ionic currents, and *I*_stim_ is the externally applied stimulus current. *C*_*m*_ is the membrane capacitance.

The cell model of heart failure (HF) used in this study was based on Elshrif et al.’s research (Elshrif et al., 2015), where a collection of HF-induced ion channel remodeling effects were incorporated into the ORd model. Similarly, the effects of CORM-2 (i.e., a CO-releasing molecule) were modeled based on previous research by Al-Owais et al. (Al-Owais et al., 2021) and were incorporated into the healthy and HF cell models. The reason we chose CORM-2 rather than CO is that CORM-2 is one of the most common CO-releasing molecules in biological research, and is safer and more controllable than CO. More details can be found in Sections SII and SIII in the Supplementary Material.

### 2.2 Modeling the effects of ranolazine and HW-0168 on ion channels

Available experimental data regarding the effects of ranolazine and HW-0168 from previous studies have been summarized in Table 1 (Antzelevitch et al., 2004; Rajamani et al., 2008; Beyder et al., 2012; Moreno et al., 2013; Dong et al., 2019). Specifically, ranolazine has been shown to exert dose-dependent blocking effects on *I*_Na_ (Beyder et al., 2012), *I*_NaL_ (Antzelevitch et al., 2004), *I*_NaCa_ (Antzelevitch et al., 2004), *I*_CaL_ (Antzelevitch et al., 2004), *I*_Kr_ (Rajamani et al., 2008). Dose-response curves for ranolazine-affected ion channels were fitted using the following Hill functions:

**TABLE 1:** Summary of data for ranolazine and HW-0168.

		*I*_Na_	*I*_NaL_	*I*_NaCa_	*I*_CaL_	*I*_Kr_
Ranolazine	IC_50_ (μM)	53.6	6.23	91	296	12
	Hill	2.4	1	1.48	1	1
	Species	HEK 293	Canine	Canine	Canine	HEK 293
	Ref	Beyder et al. (2012)	Moreno et al. (2013)	Antzelevitch et al. (2004)	Antzelevitch et al. (2004)	Rajamani et al. (2008)
HW-0168	EC_50_ (μM)	n/a	n/a	n/a	n/a	0.41
	Hill	n/a	n/a	n/a	n/a	0.73
	Act_max_	n/a	n/a	n/a	n/a	2.8
	Species	n/a	n/a	n/a	n/a	HEK 293
	Ref	n/a	n/a	n/a	n/a	Dong et al. (2019)

***I***_**Na**_

$$f_{Na}^{\mathrm{RAN}}=\frac{1.0}{1.0+{\left(\left[\mathrm{RAN}\right]/53.6\right)}^{2.4}}$$ (2)

***I***_**NaL**_

$$f_{NaL}^{\mathrm{RAN}}=\frac{1.0}{1.0+{\left(\left[\mathrm{RAN}\right]/6.23\right)}^{1.0}}$$ (3)

***I***_**NaCa**_

$$f_{NaCa}^{\mathrm{RAN}}=\frac{1.0}{1.0+{\left(\left[\mathrm{RAN}\right]/91.0\right)}^{1.48}}$$ (4)

***I***_**CaL**_

$$f_{CaL}^{\mathrm{RAN}}=\frac{1.0}{1.0+{\left(\left[\mathrm{RAN}\right]/296.0\right)}^{1.0}}$$ (5)

***I***_**Kr**_

$$f_{Na}^{\mathrm{RAN}}=\frac{1.0}{1.0+{\left(\left[\mathrm{RAN}\right]/12.0\right)}^{1.0}}$$ (6)

where [*RAN*] is the dose of ranolazine used in experiments.

**FIGURE 1:**
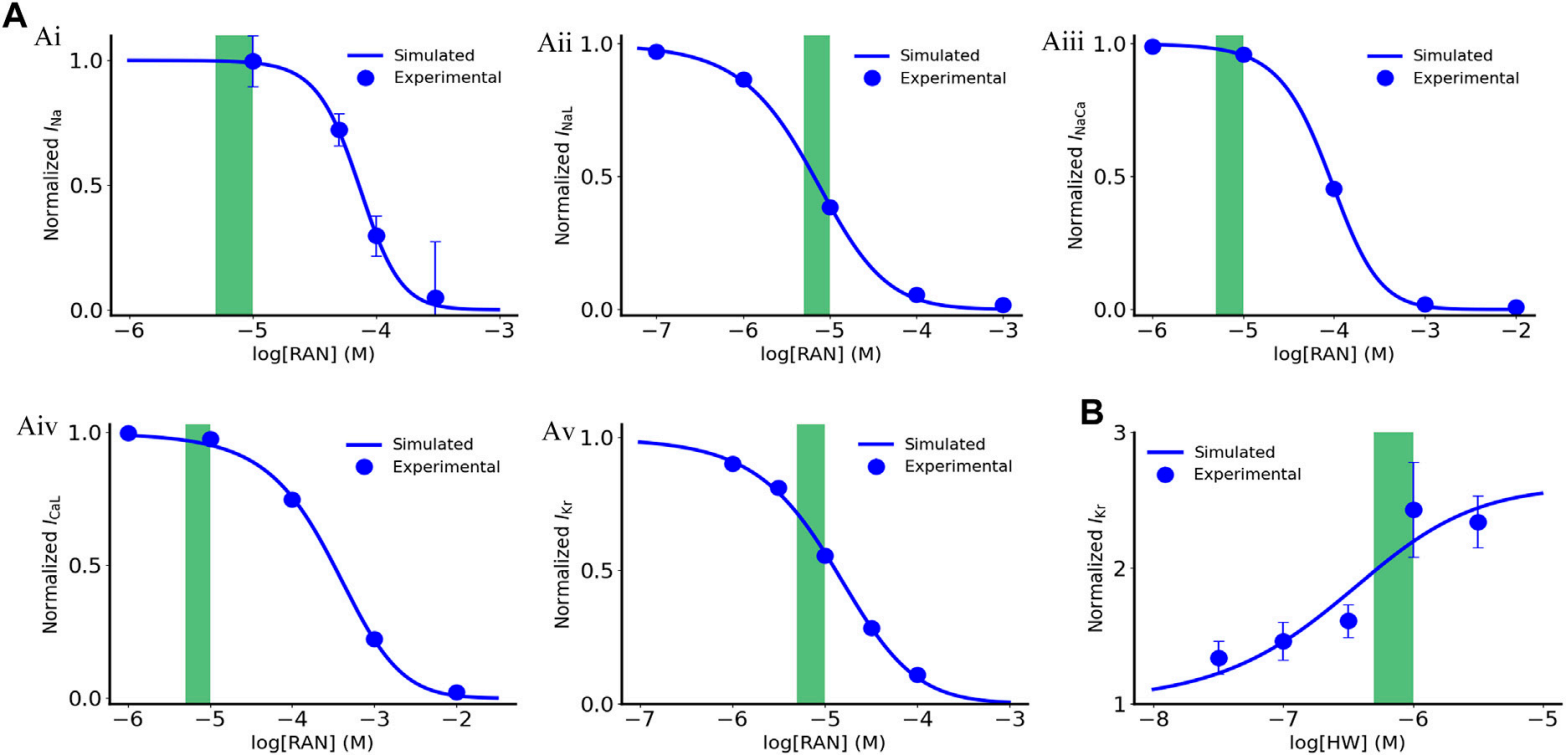
Dose dependent effects of ranolazine and HW-0168 on ionic currents. **(A)** Effects of ranolazine on *I*_Na_, *I*_NaL_, *I*_NaCa_, *I*_CaL_ and *I*_Kr_. **(B)** Effects of HW-0168 on *I*_Kr_. Green boxes indicate therapeutic ranges of ranolazine (5–10 μM) and HW-0168 (0.5–1 μM).

The fitting results are illustrated in Figure 1A. For the *I*_Kr_ activator, HW-0168, only *I*_Kr_ was reported to be affected by the drug (Dong et al., 2019); therefore, the data were fitted using Eq. 7:

*I*_Kr_

$$f_{Kr}^{HW}=\frac{1.8}{1.0+{\left(0.41/\left[HW\right]\right)}^{0.73}}+1$$ (7)

where [*HW*] is the dose of HW-0168 used in the experiment.

The fitted dose-dependent curve is illustrated in Figure 1B. In this study, we used 10 μM and 0.5 μM for ranolazine and HW-0168, respectively. The above-fitted equations were finally incorporated into the ‘Healthy + CO’ and ‘HF + CO’ cell models.

The ionic current under the action of the drug is calculated by Eq. 8:

$$I_{ion}^{Drug}=I_{ion}\cdot f_{ion}^{Drug}$$ (8)

where $f_{ion}^{Drug}$ represent the effect of a drug on a certain ionic current.

### 2.3 Simulating the efficacy of multi-channel blockers and specific *I*_Kr_ channel activators

In addition to ranolazine and HW-0168, we also selected six multi-channel blockers (i.e., amiodarone, verapamil, nifedipine, quinidine, vanoxerine, and bepridil) and four specific *I*_Kr_ activators (i.e., KB130015, ICA-105574, NS1643, NS3623) for efficacy simulation and screening of the drugs. A simple pore block theory (Brennan et al., 2009) was used in this study to model the interactions between drugs and ion channels. Based on this theory, the effect of drugs blocking ion channels was fitted by the following formula:

$$\theta =\frac{1}{1+{\left(IC_{50}/\left[D\right]\right)}^{nH}}$$ (9)

where *θ* is the blocking efficiency, [*D*] is the concentration of a drug, *IC*_*50*_ is the half-maximal inhibitory concentration, and *nH* is the Hill coefficient.

The effect of drugs activating ion channels was fitted by Eq. 11:

$$Y=\frac{{Act}_{\max}-1}{1+{\left(EC_{50}/\left[D\right]\right)}^{nH}}$$ (10)

where *Y* is the activation efficiency, and *Act*_*max*_ is the maximum activation efficiency, *EC*_*50*_ is the compound concentration resulting in 50% of the *Act*_*max*_.

The six multi-channel blockers act on related ion channels in a dose-dependent manner, and the related parameters are listed in Table 2. To evaluate the drug efficacy more objectively, we explored all drugs at three doses based on their C_max_, as shown in Table 3. The four specific *I*_Kr_ activators activated *I*_Kr_ currents in a dose-dependent manner as well, and the relevant parameters are shown in Table 4.

**TABLE 2:** Summary of data for six multi-channel blockers.

Drug		*I*_Na_	*I*_NaL_	*I*_CaL_	*I*_to_	*I*_Kr_	*I*_K1_	*I*_Ks_	*I*_NaK_	*I*_NaCa_
Amiodarone	IC_50_ (μM)	40.4	9	5.8	n/a	0.03	n/a	3.84	15.6	3.3
	Hill	0.75	0.4	1	n/a	1	n/a	0.63	1	1
	Species	Rabbit	MANTA*	Guinea pig	n/a	HEK-293	n/a	Guinea pig	Rabbit	Guinea pig
	Ref	Suzuki et al. (2013)	Sutanto et al. (2019)	Nishimura et al. (1989)	n/a	Mirams et al. (2011)	n/a	Zankov et al. (2005)	Gray et al. (1998)	Watanabe and Kimura, (2000)
Verapamil	IC_50_ (μM)	7.221	6.094	0.0794	n/a	0.831	9.033	65.587	n/a	n/a
	Hill	0.95	1.24	0.69	n/a	1.17	1	0.92	n/a	n/a
	Species	HEK 293	HEK 293	CHO cell	n/a	HEK 293	HEK 293	HEK 293	n/a	n/a
	Ref	Obejero-Paz et al. (2015)	Obejero-Paz et al. (2015)	Obejero-Paz et al. (2015)	n/a	Obejero-Paz et al. (2015)	Obejero-Paz et al. (2015)	Obejero-Paz et al. (2015)	n/a	n/a
Nifedipine	IC_50_ (μM)	56.2	n/a	0.3	26.8	275	260	360	n/a	n/a
	Hill	0.59	n/a	1	0.97	0.9	0.85	0.97	n/a	n/a
	Species	Human	n/a	Guinea pig	Human	Guinea pig	Guinea pig	Guinea pig	n/a	n/a
	Ref	Li et al. (2009)	n/a	Shen et al. (2000)	Gao et al. (2005)	Zhabyeyev et al. (2000)	Zhabyeyev et al. (2000)	Zhabyeyev et al. (2000)	n/a	n/a
Quinidine	IC_50_ (μM)	17	12	14.9	21.8	0.41	42.6	44	n/a	n/a
	Hill	0.92	1	1.1	0.67	0.76	0.25	1.8	n/a	n/a
	Species	Guinea pig	Rabbit	Guinea pig	Human	HEK 293	Human	CHO cell	n/a	n/a
	Ref	Koumi et al. (1992)	Wu et al. (2008)	Zhang and Hancox, (2002)	Nenov et al. (1998)	Paul et al. (2002)	Nenov et al. (1998)	Kang et al. (2001)	n/a	n/a
Vanoxerine	IC_50_ (μM)	0.0346	0.0852	0.0162	2	0.0093	98.142	2.9	n/a	n/a
	Hill	0.97	1.62	0.63	1	1.11	1	1	n/a	n/a
	Species	HEK 293	HEK 293	CHO cell	Mouse L cells	HEK 293	HEK 293	CHO cell	n/a	n/a
	Ref	Obejero-Paz et al. (2015)	Obejero-Paz et al. (2015)	Obejero-Paz et al. (2015)	Lacerda et al. (2010)	Obejero-Paz et al. (2015)	Obejero-Paz et al. (2015)	Lacerda et al. (2010)	n/a	n/a
Bepridil	IC_50_ (μM)	0.517	0.411	0.157	n/a	0.0738	66.536	6.156	n/a	n/a
	Hill	1.14	1.72	1.08	n/a	1.33	1	2.33	n/a	n/a
	Species	HEK 293	HEK 293	CHO cell	n/a	HEK 293	HEK 293	HEK 293	n/a	n/a
	Ref	Obejero-Paz et al. (2015)	Obejero-Paz et al. (2015)	Obejero-Paz et al. (2015)	n/a	Obejero-Paz et al. (2015)	Obejero-Paz et al. (2015)	Lacerda et al. (2010)	n/a	n/a

*MANTA, the Maastricht Antiarrhythmic Drug Evaluator, integrated published computational cardiomyocyte models from different species, regions and disease conditions. #The drugs in the table are all inhibitory for the channels listed, so their effects are not individually marked in the table.

**TABLE 3:** C_max_ and experimental dose allocation for six multi-channel blockers.

	Amiodarone	Verapamil	Nifedipine	Quinidine	Vanoxerine	Bepridil
C_max_ (μM)	0.0001–0.0005	0.025–0.081	0.0031–0.0077	0.924–3.237	0.00088–0.00753	0.01–0.033
Ref	Mirams et al. (2011)	Mirams et al. (2011)	Mirams et al. (2011)	Mirams et al. (2011)	Hagiwara-Nagasawa et al. (2021)	Mirams et al. (2011)
High dose (μM)	0.005	0.3	0.05	10	0.05	0.1
Medium dose (μM)	0.0005	0.03	0.005	1	0.005	0.01
Low dose (μM)	0.00005	0.003	0.0005	0.1	0.0005	0.001

**TABLE 4:** Summary of data for four specific *I*_Kr_ activators.

*I*_Kr_ activators	KB130015	ICA-105574	NS1643	NS3623
EC_50_ (μM)	12.2	0.42	10.4	79.4
Hill	1.1	2.5	1.8	1.3
Act_max_	4.7	5.5	1.5	2.9
Species	HEK 293	HEK 293	Xenopous oocytes	Xenopous oocytes
Ref	Gessner et al. (2010)	Asayama et al. (2013)	Casis et al. (2006)	Hansen et al. (2006)

### 2.4 Modeling the conduction of action potentials in one-dimensional (1D) transmural ventricular strands

The 1D transmural ventricular strand model, which is a linear syncytium formed by coupling multiple cells, can be calculated by adding a diffusion term to the cell model equation:

$$\frac{\partial V_{m}}{\partial t}=D\left(\frac{\partial^{2}V_{m}}{\partial x^{2}}\right)-\frac{I_{ion}}{C_{m}}$$ (11)

where *D* is the scalar diffusion coefficient that decides the conduction velocity of APs.

The 1D transmural strand was 15 mm long, which was close to the normal range of the human transmural ventricle width (∼4.0–14.0 mm) (Drouin et al., 1995; Yan et al., 1998). The strand was discretized into 100 interconnected nodes with a spatial precision of 0.15 mm, which was consistent with the reported cell length [i.e., 80–150 μm (Hinrichs et al., 2011)]. The proportions for transmural cell types were set to 25:35:40 for ENDO, MID, and EPI cells, which were identical to that used in previous studies (Zhang and Hancox, 2004; Luo et al., 2017). Such proportions reliably reproduced a positive T wave in the computed pseudo-ECG under control (healthy) conditions. The diffusion coefficient *D* was set to 0.127 mm^2^/ms, giving a CV of planar excitation waves of 70 cm/s through the strand, which matched well with the experimental data from human ventricles (Taggart et al., 2000).

### 2.5 Modeling the conduction of action potentials in the 1D strand with CO-affected regions

**FIGURE 2:**
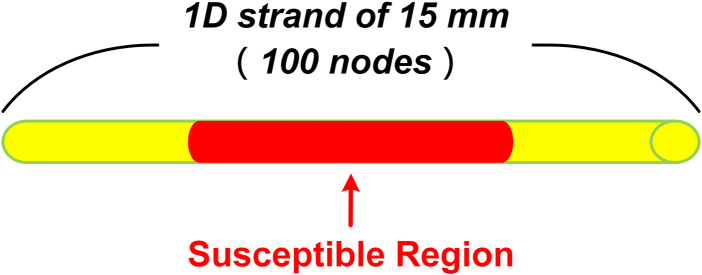
Schematic of 1D homogenous ventricular strand model with CO-affected regions. The tissue is 15 mm in length and contains 100 cells. The red part in the middle indicates the susceptible region where the number of cells varies from 0 to 100.

To further quantify the critical size of EAD cells for overcoming the source-sink effect and initiating triggers in ventricular tissue, we simulated a 15 mm homogenous ventricular strand consisting of only MID cells for the failing heart, with the center of the strand (Figure 2, red region) containing a variable number of contiguous cells affected by CO. The number of cells in the susceptible region was gradually increased until the synchronously occurred EADs overcame the source-sink effect and trigger a premature beat. The critical cell number was recorded as a metric for measuring the susceptibility to arrhythmias.

### 2.6 Generating pseudo-ECGs using the 1D model

The pseudo-ECG was calculated from the constructed 1D strand model by the following equation (Gima and Rudy, 2002):

$$\phi_{e}\left(x^{\prime }\right)=\frac{a^{2}}{4}\int \left(-\nabla V_{m}\right)\cdot \left[\nabla \frac{1}{r}\right]dx$$ (12)

where $\phi_{e}$ is a unipolar potential generated by the strand, *a* is the radius of the strand, *dx* is the spatial resolution, and *r* is the Euclidean distance from a point *x* to another point *x′*.

**FIGURE 3:**
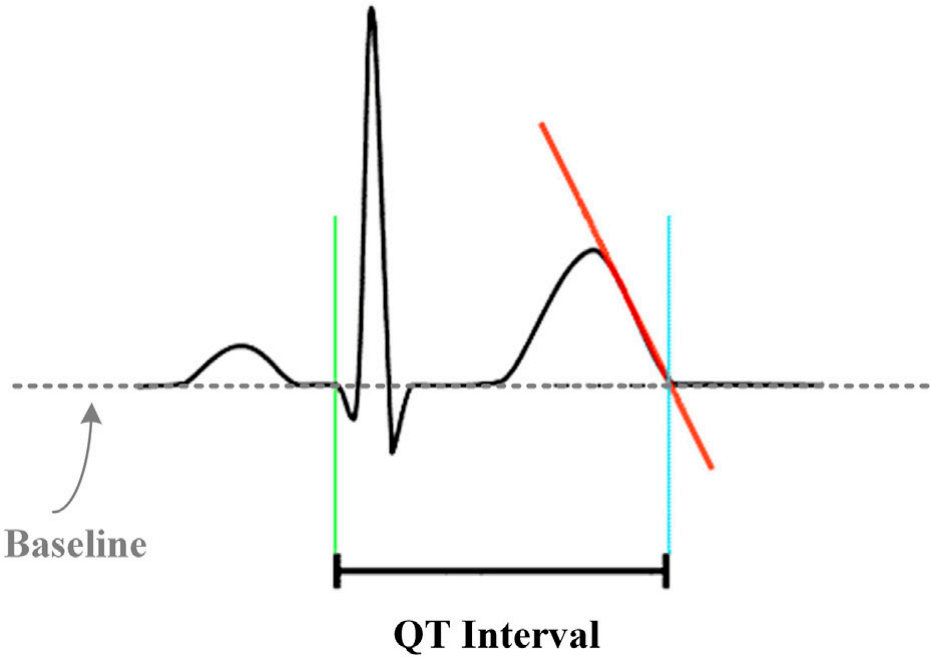
Schematic diagram of the QT interval measuring method.

As shown in Figure 3, the period from the earliest appearance of the QRS complex to the end of the T-wave was defined as the QT interval, measured in milliseconds. The end of the T-wave was defined as the return of the descending limb to the TP baseline.

### 2.7 Modeling cell populations

To demonstrate the robustness of the reported findings, we constructed cell population models with reference to previous studies (Britton et al., 2013; Sutanto and Heijman, 2020). Specifically, the maximum conductance of the nine major ionic currents (*I*_Na_, *I*_NaL_, *I*_CaL_, *I*_Kr_, *I*_Ks_, *I*_K1_, *I*_to_, *I*_NaCa_, and *I*_NaK_) in the original deterministic model was scaled by a group of factors that follow a normal distribution with mean 1.0 and standard deviation 0.2. In this way, 1,000 population model variants were obtained.

### 2.8 Dynamic restitution protocol

The CV dynamic restitution curves were obtained using a dynamic pacing protocol. Specifically, the 1D strand model was paced with a certain basic cycle length (BCL) until reading its steady state upon which the CV value was recorded for that BCL. The initial BCL was set to 3,000 ms and was decreased gradually until the model failed to produce excitation waves. Based on the ‘CV-BCL’ pairs generated by the above protocol, CV restitution curves could be plotted against BCL.

### 2.9 Modeling the conduction of excitation waves on a two-dimensional (2D) realistic ventricular slice

Similar to the 1D strand model, the monodomain equation (Eq. 11) was adopted to describe the propagation of excitation waves in the ventricular slice. Isotropic propagation was assumed, and the diffusion coefficient D was set to 0.154 mm^2^/ms, to produce a CV of 0.74 m/s (Taggart et al., 2000). The spatial step was set to 0.15 mm to be consistent with that in 1D models. To mimic the physiological characteristics of the Purkinje fibers, a series of supra-threshold stimuli were applied to several pacing sites on the endocardium of the slice.

## 3 Results

### 3.1 Assessing the drug efficacy of multi-channel blockers on CO-affected hearts

#### 3.1.1 Effects of ranolazine on AP and ECG

**FIGURE 4:**
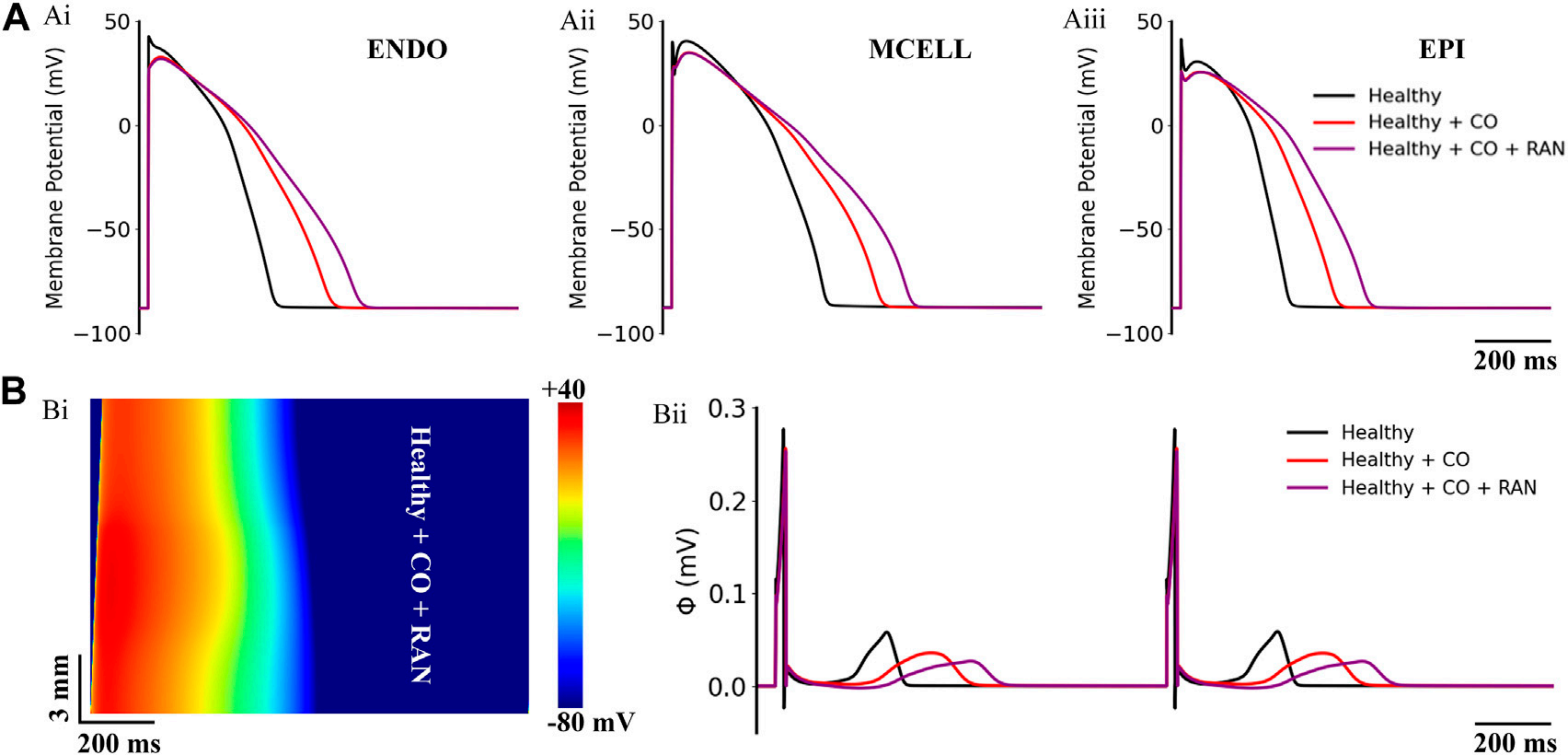
Actions of ranolazine (RAN) on CO-affected myocardial cells and tissues. **(A)** The comparison of action potentials of three cell types under ‘healthy’, ‘healthy + CO’, and ‘healthy + CO + RAN’ conditions. **(B)** Spatial-temporal plots under the ‘healthy + CO + RAN’ condition (Bi), and the corresponding pseudo-ECG (Bii).

Previous studies have suggested the drug ranolazine to be a potential pharmacotherapy for the treatment of CO-induced arrhythmias (Dallas et al., 2012). Therefore, we first tested the efficacy of ranolazine on the baseline model of ‘healthy + CO’. Simulation results are illustrated in Figure 4. Interestingly, ranolazine aggravated the arrhythmogenesis of CO. At the cellular level, it can be observed that ranolazine (10 μM) further extended APDs of all cell types, and APD_90_ values of ENDO, MID, and EPI cells were increased by 15.7%, 14.6%, and 20.3% based on CO conditions, respectively (Figure 4A). At the tissue level, generated pseudo-ECGs using 1D transmural ventricular strand models showed that ranolazine further prolonged the QT interval and decreased the T-wave amplitude (Figure 4Bii). The effect of ranolazine was also reflected in conduction properties, where the tissue with ranolazine owned a wider wavelength (Figure 4Bi) than the control condition.

#### 3.1.2 Effects of six multi-channel blockers on ECG

**FIGURE 5:**
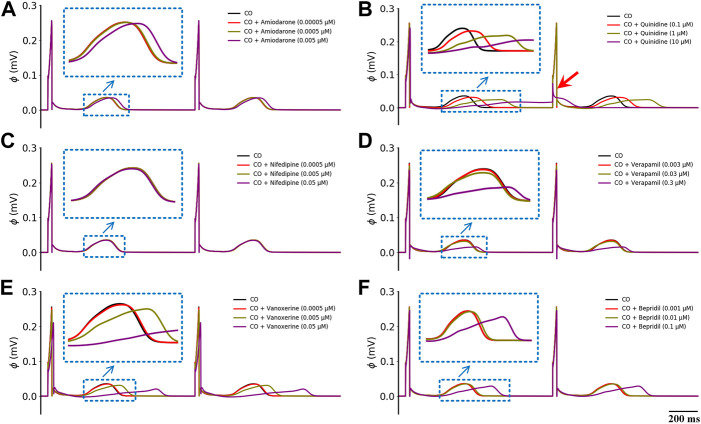
Effects of six multi-channel blockers at three doses on ECG morphology under healthy conditions. **(A)** amiodarone, **(B)** quinidine, **(C)** nifedipine, **(D)** verapamil, **(E)** vanoxerine and **(F)** bepridil. Blue ‘↓’ indicates the magnification of the rectangular area; red ‘↓’ indicates the failed depolarization in ECG.

To find out if there are any available medications for the treatment of CO-induced arrhythmias, we collected the experimental data regarding the blocking effects of drugs on various channels as possible (see Table 2 in the *Method* section), and incorporated them into the baseline model to explore their potential treatment to CO-induced arrhythmias. In this study, three experimental doses were designed based on the C_max_ of these drugs (as shown in Table 3). The simulated pseudo-ECGs are shown in Figure 5.

It can be observed that all six drugs failed to restore the prolonged QT interval even at their ‘high’ doses that are remarkably higher than the C_max_ level (i.e., ‘high dose’ = 10×C_max_). Specifically, low doses of amiodarone, nifedipine, verapamil, vanoxerine, and bepridil had no effects on the QT interval, while a low dose of quinidine exerted mild QT prolongation effects. When moderate doses were applied, quinidine and vanoxerine considerably prolonged the QT interval, while the other drugs still had no sensible effects. Finally, at high doses, all drugs except nifedipine prolonged the QT interval to varying degrees. Among them, vanoxerine and bepridil considerably prolonged the QT interval, and quinidine led to ECG repolarization failure.

#### 3.1.3 Independent component analysis of ion channels

To determine the independent role of each drug-affected ion channels, we performed an ion mechanism analysis with ranolazine as a representative case. First, we quantitatively analyzed the individual role of each ion channel involved in the action of ranolazine. APD_90_ was used as the metric, and the results are summarized in Table 5. It can be observed that the effects of ranolazine on *I*_Na_, *I*_NaCa_, and *I*_CaL_ have no effect on APD_90_. On the other hand, the inhibition effect of ranolazine on *I*_NaL_ shortened the APD_90_ of all three cell types, demonstrating an antiarrhythmic action; however, the simultaneously inhibited *I*_Kr_ by ranolazine led to a more pronounced prolonging of APD, which offset the effects of *I*_NaL_ and aggravated the CO-induced arrhythmogenesis at the cellular level.

**TABLE 5:** Effects of ranolazine-induced changes in single ion channels on APD_90_.

Ion channels	*I*_Na_	*I*_NaL_	*I*_NaCa_	*I*_CaL_	*I*_Kr_
APD_90_ (ENDO)	0	9.4%↓	0	0	27.1%↑
APD_90_ (MID)	0	7.0%↓	0	0	24.1%↑
APD_90_ (EPI)	0	4.4%↓	0	0	26.3%↑

‘↑’ and ‘↓’indicate that the effect of the change of this ion channel on APD90 is lengthening or shortening.

**FIGURE 6:**
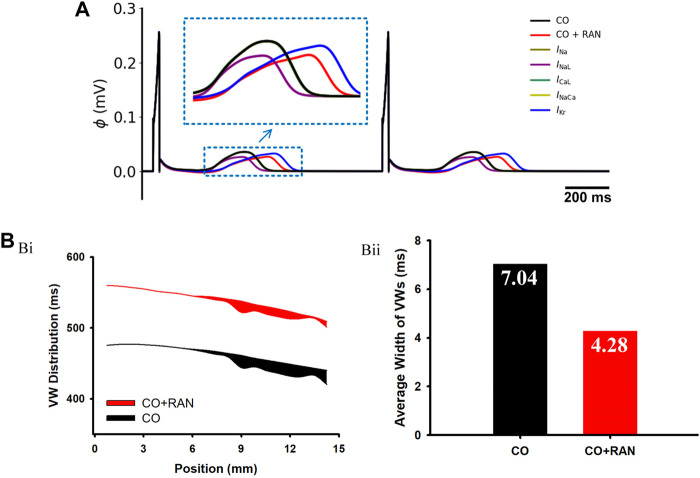
Simulation results of ranolazine single-channel analysis and Vulnerable Window (VW) in a 1D transmural ventricular strand model. **(A)** Pseudo-ECG under the single-channel effect of ranolazine. **(B)** Simulation results for VW. (Bi) Distribution of VWs across the strand. Black and red belts stand for the ‘CO’ and ‘CO + ranolazine’ conditions, respectively. (Bii) Comparisons of the average width of VWs in the two conditions.

Next, we analyzed the individual role of each ion channel in the ECG changes, as shown in Figure 6A. Consistent with the results at the cellular level, the effects of ranolazine on *I*_Na_, *I*_NaCa_, and *I*_CaL_ did not cause any obvious ECG changes. More specifically, the IC_50_ values of ranolazine for *I*_Na_, *I*_NaCa_, and *I*_CaL_ were 53.6 μM, 91.0 μM, and 296.0 μM, and ranolazine at 10 μM inhibited only 1.8%, 3.7%, and 3.3% of *I*_Na_, *I*_NaCa_, and *I*_CaL_, respectively, which had almost no effect on APD and ECG. As for the *I*_NaL_, the QT interval shortening effect caused by the inhibition of *I*_NaL_ could not offset the QT interval prolongation by the attenuation of *I*_Kr_. So overall, ranolazine eventually led to QT prolongation.

#### 3.1.4 Effects of drugs on the transmural dispersion of repolarization

In this part, we assessed the role of heterogeneity among different ventricular cells on arrhythmias. Simulations at the cellular level show that, under the action of ranolazine, the APD difference between MID and ENDO cells (ΔAPD_MID-ENDO_) decreased from 63 ms to 61 ms, and ΔAPD_MID-EPI_ reduced from 111 ms to 109 ms. The decreased ΔAPD among different cell types suggested that the drug decreased the vulnerability in terms of transmural heterogeneity. The following experiments of vulnerable window measurements using transmural 1D strand further confirmed this. As shown in Figures 6Bi,Bii, the average width of the VW under the ‘CO + RAN’ condition is apparently narrower compared to that in the CO condition (from 7.04 ms to 4.28 ms). The decreased temporal risk evidenced by the vulnerable window changes is consistent with the cellular level simulation results.

#### 3.1.5 Effects of drugs on conduction velocity

**FIGURE 7:**
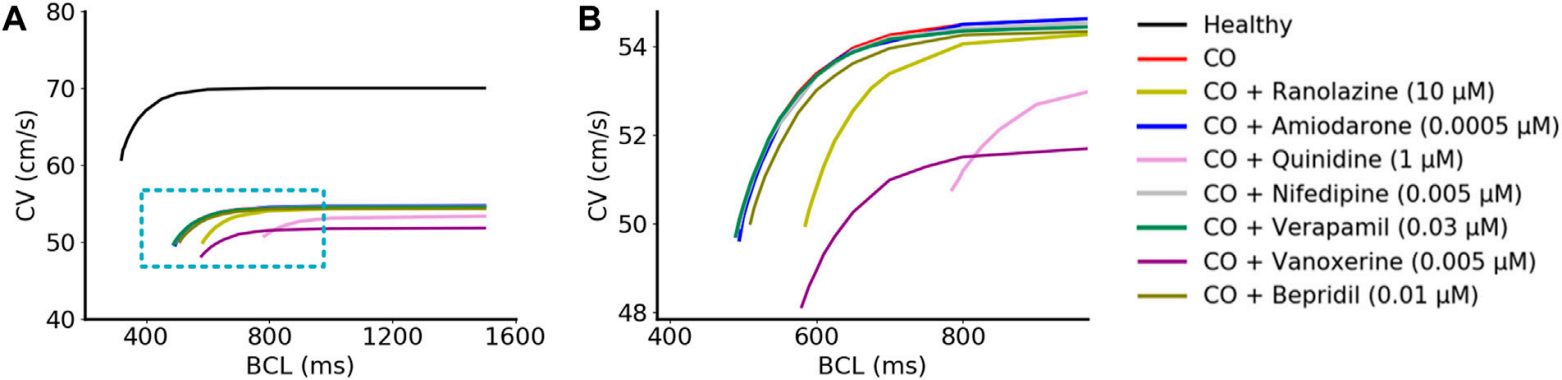
Simulated CV restitution curves in different conditions. **(A)** CV restitution curves under ‘healthy’, ‘CO’, and ‘CO + drug’ conditions. The magnified view inside the blue rectangle was shown in **(B)**.

Simulations demonstrated that the CV under ‘CO’ and ‘CO + drug’ conditions were lower for all BCLs compared to the healthy conditions (Figure 7A). Specifically, after the addition of amiodarone, verapamil, nifedipine, and bepridil, the CV dynamic restitution curves were almost unchanged compared to CO conditions, suggesting that amiodarone, verapamil, nifedipine, and bepridil had no effect in terms of the tissue conduction properties (Figure 7B). Vanoxerine caused a further decrease in CV on the basis of CO, and ranolazine led to a right shift of the CV curve and an increase in the curve slope. Quinidine caused a mild decrease in CV and impaired the adaptability of tissue to fast heart rates (small BCLs).

In general, none of these drugs could restore the decreased CV by CO, and some of them even aggravated this situation. Furthermore, the decreased CV also contributed to a smaller wavelength (calculated as CV×ERP) and might therefore help to maintain the reentrant waves within a limited tissue size.

### 3.2 Assessing the drug efficacy of multi-channel blockers on CO-affected hearts accompanied by heart failure

**FIGURE 8:**
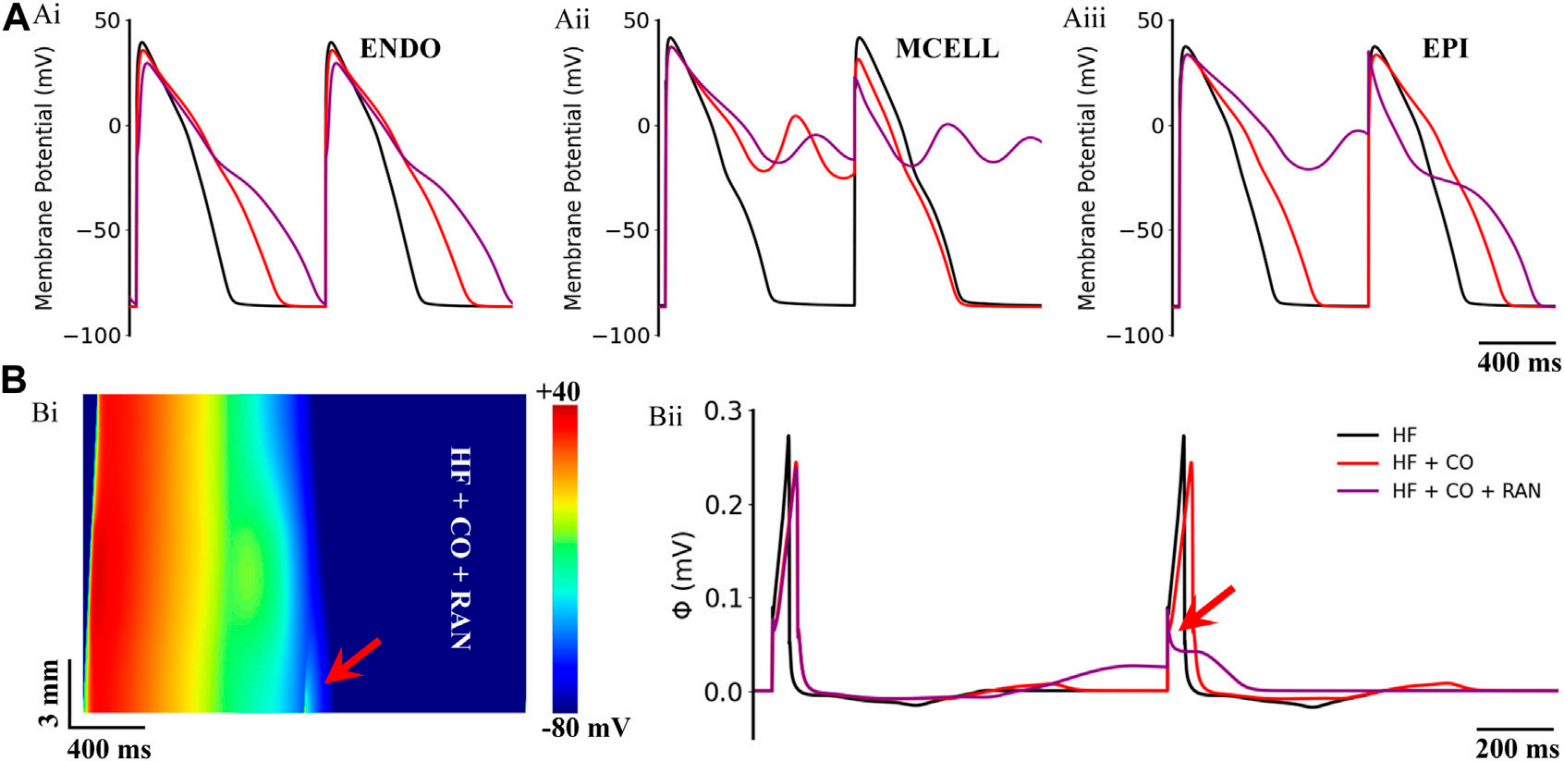
Actions of ranolazine (RAN) on CO-affected myocardial cells and tissues accompanied by heart failure (HF). **(A)** The comparison of action potentials of three cell types under ‘HF’, ‘HF + CO’, and ‘HF + CO + RAN’ conditions. **(B)** Spatial-temporal plots under the ‘HF + CO + RAN’ condition (Bi), and the corresponding pseudo-ECGs (Bii).

The influences of the aforementioned drugs were also evaluated under the heart failure condition. Simulated actions of ranolazine on CO-affected cells and tissues of heart failure are presented in Figure 8. Overall, ranolazine exacerbated the CO and heart failure-induced arrhythmias. In detail, the CO-induced 2:1 alternated EADs in MID cells became 1:1 consecutive EADs (Figure 8Aii), resulting in complete repolarization failure. Ranolazine also led to the occurrence of EAD in EPI cells (Figure 8Aiii). Above EAD activities in single cells did not develop into ectopic beats in 1D ventricular strands due to the ‘source-sink’ effect (Xie et al., 2010); however, ranolazine resulted in the 1:1 conduction failure of excitation waves at the pacing frequency of 1.25 Hz (Figure 8Bi). For the pseudo-ECG, ranolazine did not eliminate the CO-induced ECG morphological changes in heart failure tissue and further led to failed depolarization due to the considerably prolonged repolarization phase of the last cycle (Figure 8Bii).

**FIGURE 9:**
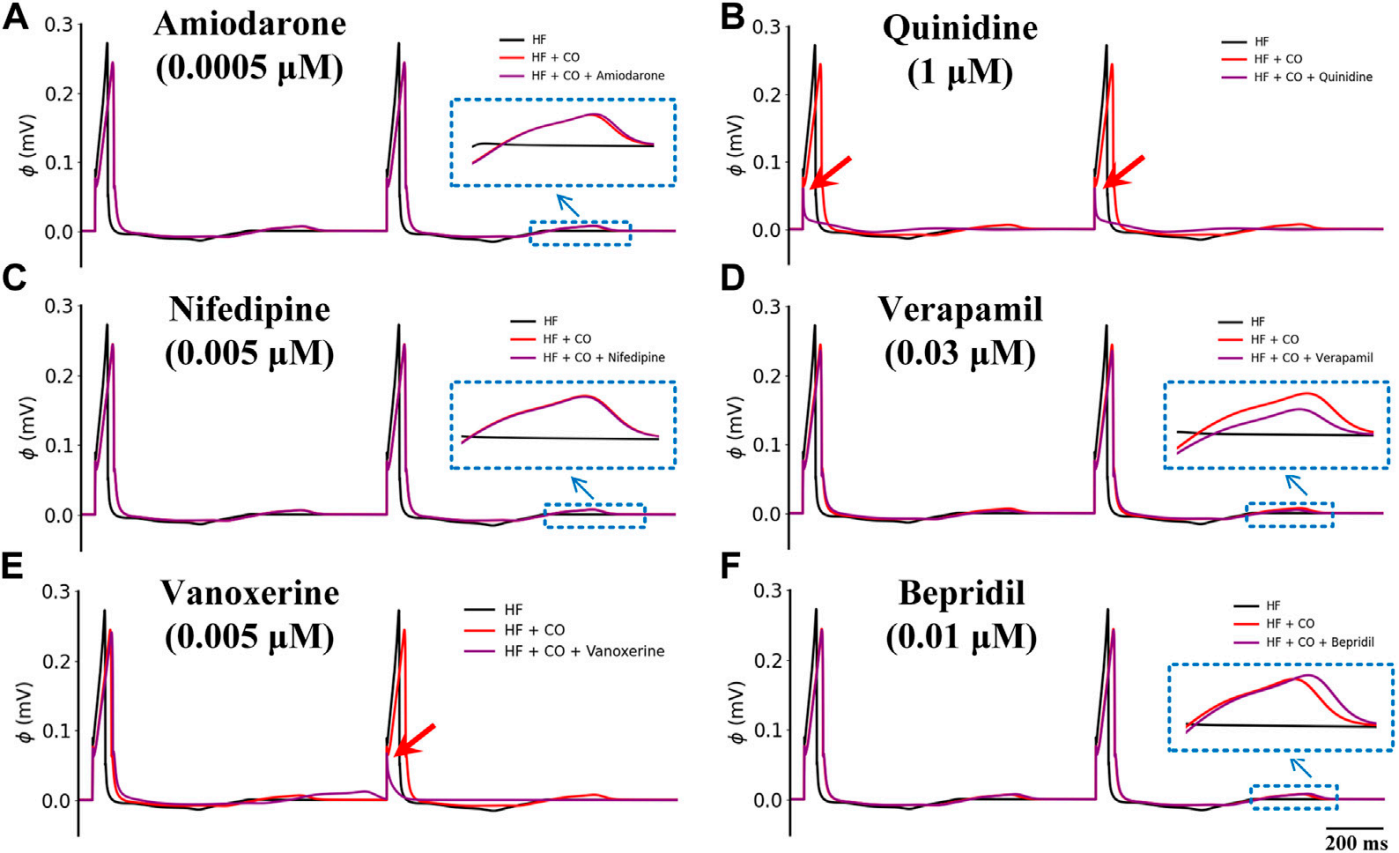
Effects of six multi-channel blockers on CO-affected ECG morphology by heart failure (HF). ECG morphology of **(A)** amiodarone (0.0005 μM), **(B)** quinidine (1 μM), **(C)** nifedipine (0.005 μM), **(D)** verapamil (0.03 μM), **(E)** vanoxerine (0.005 μM) and **(F)** bepridil (0.01 μM).

Figure 9 shows the effects of the other six multi-channel blockers on ECG morphology in heart failure conditions. Due to the remodeled transmural gradient of repolarization in the heart failure condition, the T-wave was almost flattened. In terms of the QT-interval, amiodarone (0.0005 μM), nifedipine (0.005 μM), and verapamil (0.03 μM) had almost no effect on the QT interval, and bepridil (0.01 μM) slightly prolonged the QT interval. In addition, quinidine (1 μM) and vanoxerine (0.005 μM) caused depolarization failure. Overall, all six drugs were not effective against CO-induced arrhythmias in heart failure conditions.

### 3.3 Investigating the critical cell number for triggering ectopic beats

**FIGURE 10:**
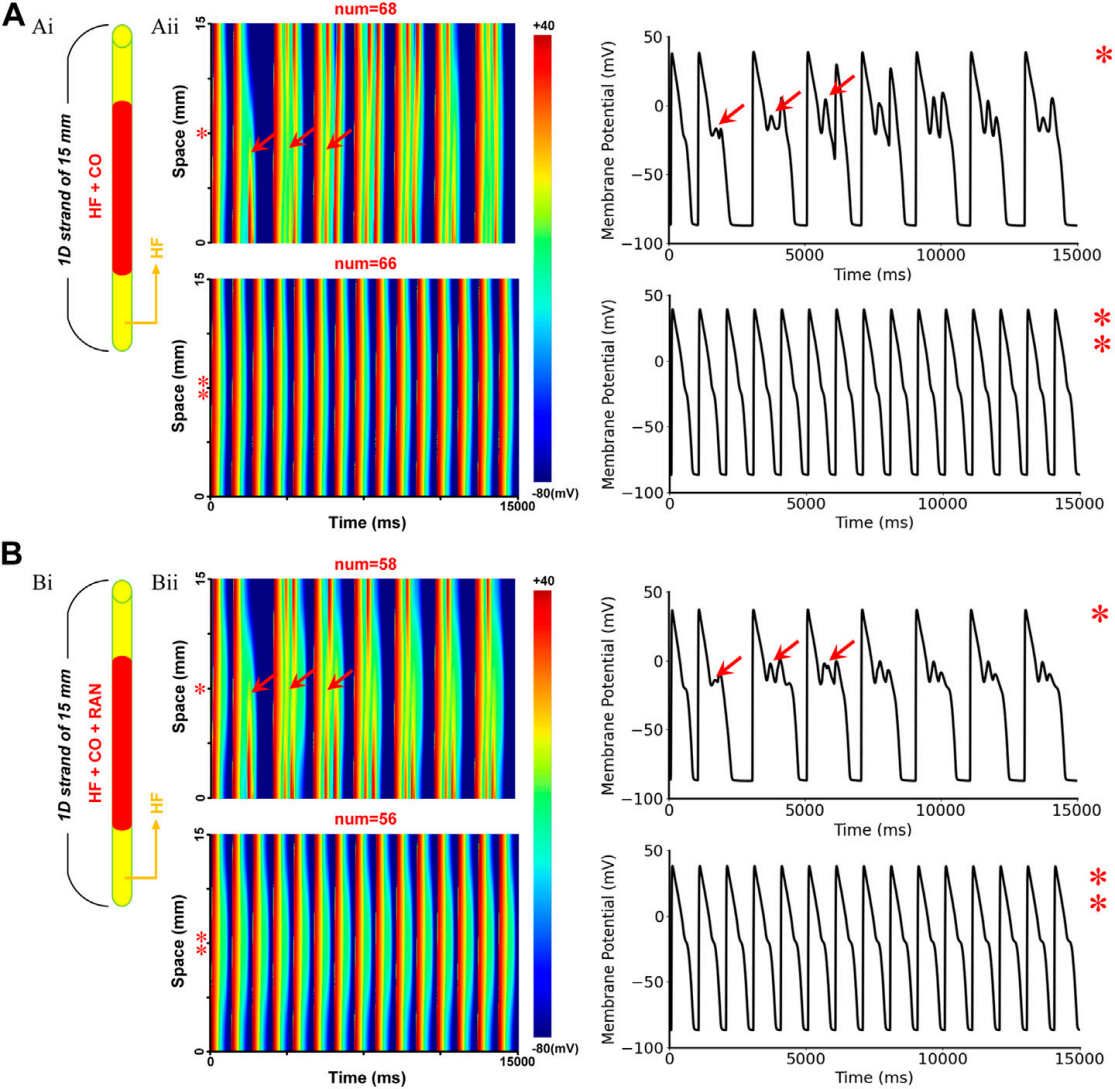
The critical size for initiating ectopic beats in failing 1D homogenous ventricular strands. **(A)** Simulated effects of CO on the failing 1D tissue: (Ai) Schematic of the model infected by CO region, the red region represents CO-affected cells, whereas the yellow regions at both ends represent cells that were not affected by CO; (Aii) Schematic of the 1D excitation wave conduction in the CO-affected tissue model (left) and the corresponding APs of the cells marked ‘*’ or ‘**’ (right). The red arrows (‘↑’) represent the location of ectopic beats and the corresponding EADs. **(B)** Simulation results under CO + ranolazine conditions.

The baseline model of HF + CO showed that CO could induce pronounced EAD activities in MID cells, but these EADs did not evolve into ectopic beats in 1-D tissue due to the ‘source-sink’ effect (i.e., the depolarization force of EAD is not able to trigger an excitation due to the limited number of EAD cells) (Xie et al., 2010). Applying ranolazine did not trigger ectopic beats in the tissue either; however, it did diminish the repolarization ability in terms of the cellular action potential (Figure 8A). To give a more intuitive presentation of the increased proarrhythmic risk of ranolazine, we quantified the risk by measuring the *critical number* for generating the ectopic beat. Specifically, we constructed a 1D model of HF MID cells, with its central segment being set to CO-affected, and the minimum number of affected cells that could overcome the source-sink effect and lead to ectopic beats was recorded as the *critical cell number*. As shown in Figure 10, simulations suggested that the critical cell number under CO conditions was 68, corresponding to a tissue length of 10.2 mm. In contrast, the critical cell number was only 58 after the addition of ranolazine, which suggested an increased susceptibility to ectopic beats. Action potentials of representative cells within the CO-affected region (marked ‘*’ and ‘**’ in Figure 10) were plotted in the right panels of Figure 10.

### 3.4 Assessing the drug efficacy of specific *I*_Kr_ activators on CO-affected hearts in healthy and concomitant heart failure

**FIGURE 11:**
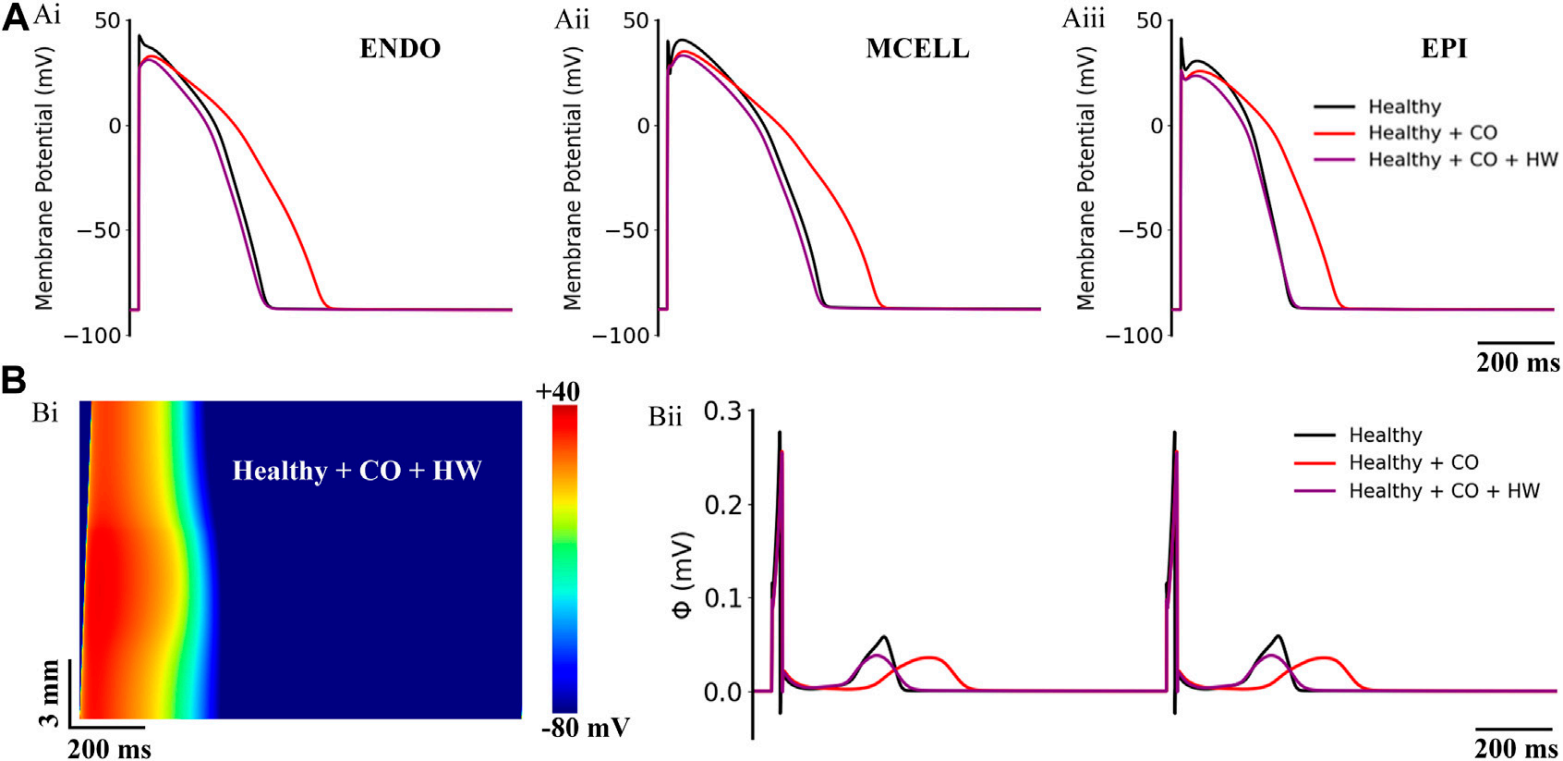
Actions of HW-0168 (HW) on CO-affected myocardial cells and tissues. **(A)** The comparison of action potentials of three cell types under ‘healthy’, ‘healthy + CO’, and ‘healthy + CO + HW’ conditions. **(B)** Spatial-temporal plots under the ‘healthy + CO + HW’ condition (Bi), and the corresponding pseudo-ECGs (Bii). Noted that the HW-0168 restored the QT interval almost to the control level.

**FIGURE 12:**
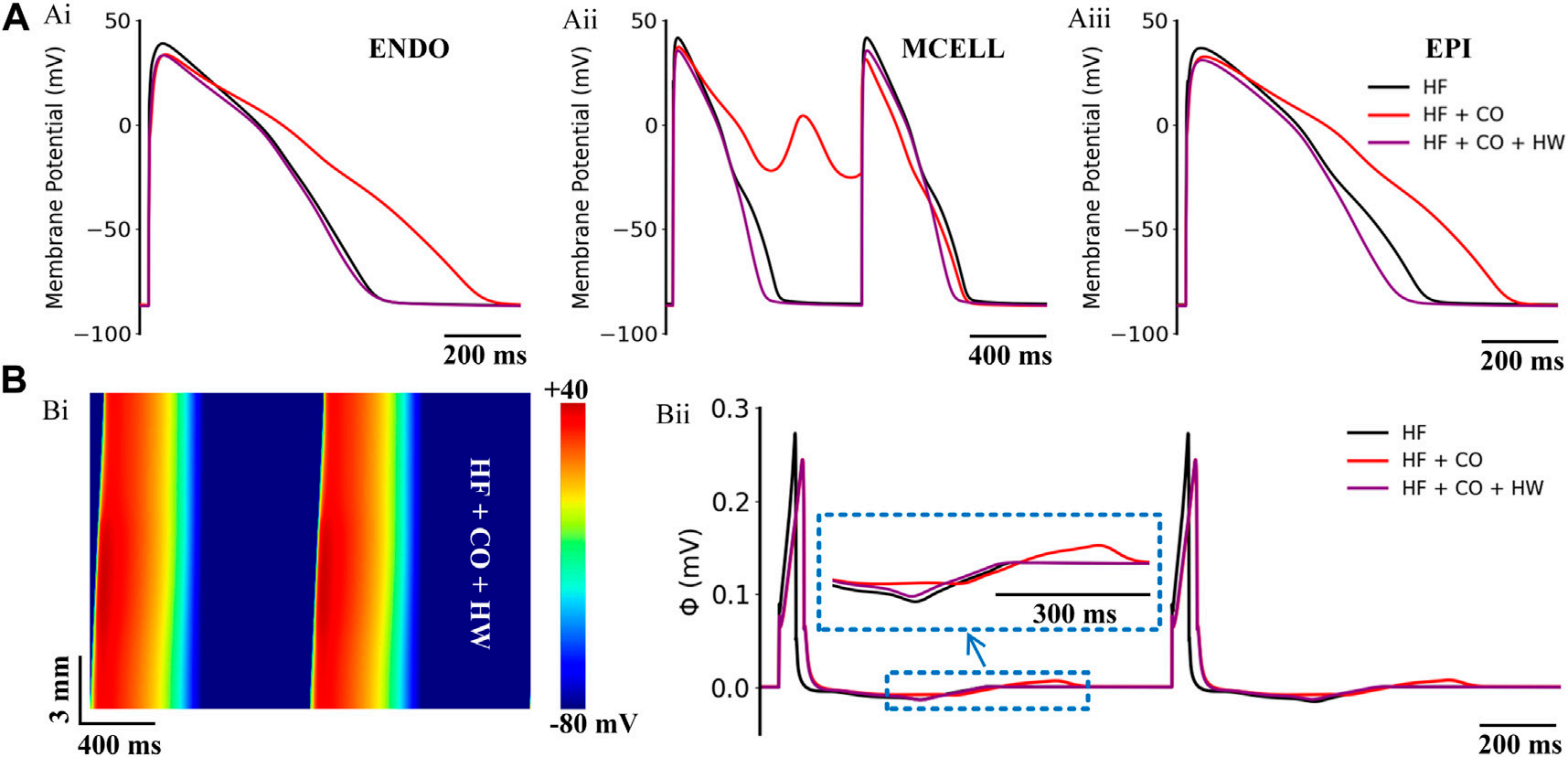
Actions of HW-0168 (HW) on CO-affected myocardial cells and tissues accompanied by heart failure. **(A)** The comparison of action potentials of three cell types under ‘HF’, ‘HF + CO’, and ‘HF + CO + HW’ conditions. **(B)** Spatial-temporal plots under the ‘HF + CO + HW’ condition (Bi), and the corresponding pseudo-ECGs (Bii).

In our previous study (Jiang et al., 2022), we have shown that the suppression of *I*_Kr_ is the main factor responsible for the CO-induced prolongation of APD and QT interval. Considering the critical role of *I*_Kr_ in the pathological pathway and the bad efficacy of multi-channel blockers, we evaluated several specific *I*_Kr_ activators in this section. For simplicity, the simulation results of a representative drug HW-0168 (full name: N-(2-(tert-butyl)phenyl)-6-(4-chlorophenyl)-4-(trifluoromethyl) nicotinamide) (Dong et al., 2019) are presented in detail (Figures 11, 12), whereas only the effective dose is recorded for the other activators (Table 4).

On the ‘healthy + CO’ condition, it can be observed that the HW-0168 at a dose of 0.5 μM [therapeutic range suggested in clinical: 0.5–1 μM (Dong et al., 2019)] effectively shortened the APD prolongation caused by CO and reversed the prolonged APD_90_ to almost the same as the healthy condition. Generated pseudo-ECGs using 1D transmural ventricular strand models showed consistent results——HW-0168 restored the prolonged QT interval to a level that was almost identical to the control condition (Figure 11Bii). In addition, HW-0168 also improved the conduction properties of excitation waves and shortened the conduction wavelength of the tissue (Figure 11Bi).

The efficacy of HW-0168 under heart failure conditions is presented in Figure 12. Simulation results showed that HW-0168 effectively reversed the proarrhythmic effects (i.e., prolonged APDs and EADs) of CO in all three cell types (Figure 12A), and shortened the excitation wavelength in the heart failure tissue (Figure 12Bi). For the ECG, although the drug did not restore the altered T-wave morphology in heart failure, it eliminated the QT interval prolongation effects by CO.

According to the above results, the selective *I*_Kr_ activator achieved desired treatment for CO-induced arrhythmias. Therefore, more existent *I*_Kr_ activators (i.e., KB130015 (Gessner et al., 2010), ICA-105574 (Asayama et al., 2013), NS1643 (Casis et al., 2006), NS3623 (Hansen et al., 2006)) were tested and the doses of drugs under which the QT-interval was restored were recorded in Table 6. According to our simulation results, ICA-105574 was the most sensitive one, which restored the QT-interval and suppressed EADs (under heart failure conditions) at a dose of only 0.25 μM.

**TABLE 6:** Simulated therapeutic doses of four specific *I*_Kr_ activators.

*I*_Kr_ activators	KB130015	ICA-105574	NS1643*	NS3623
Therapeutic dose (μM)	5	0.25	30	85

*Noted that the maximum *I*_Kr_ activation (152%) of NS1643 was still not able to restore the QT interval to its control level. However, 30 μM NS1643 greatly shortened the QT interval to a normal range and was enough to suppress EADs in heart failure cells.

### 3.5 Simulating drug efficacy based on cell population models

**FIGURE 13:**
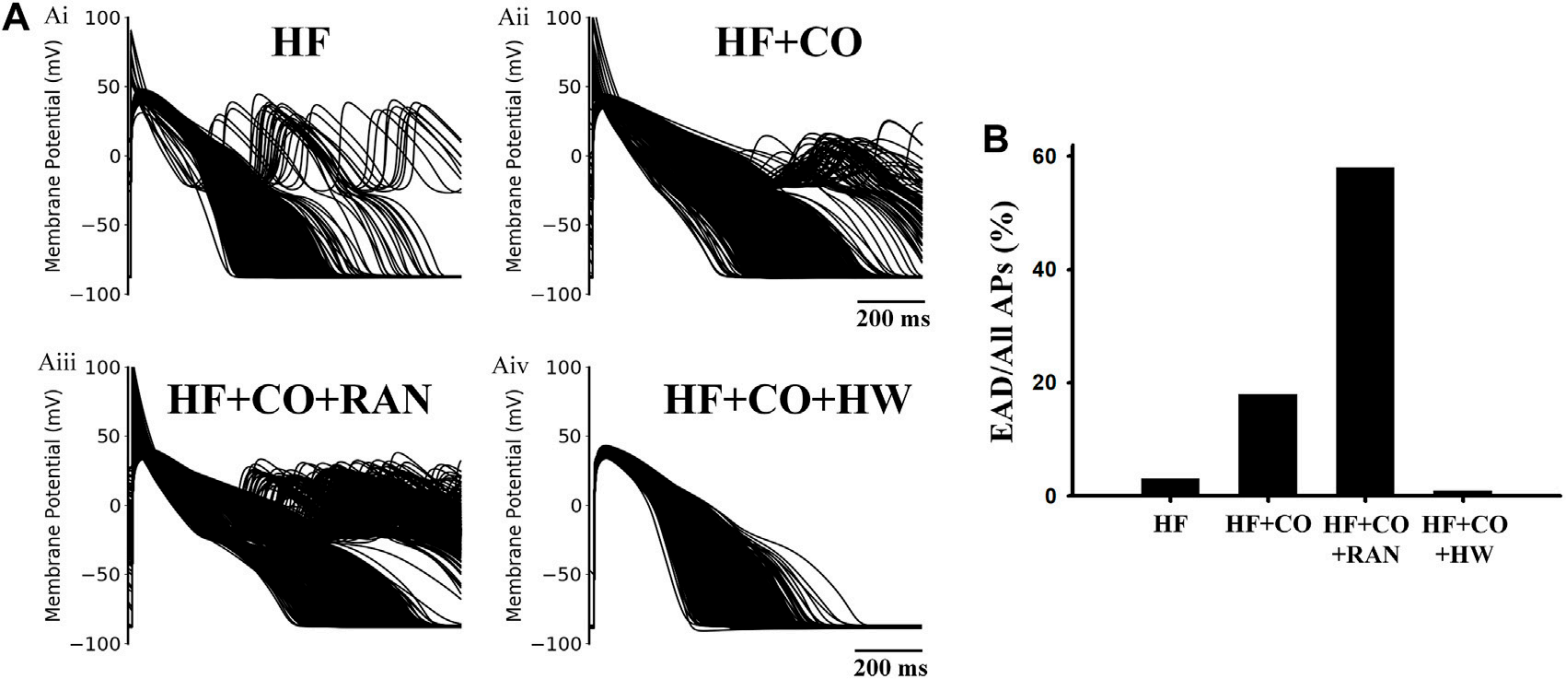
Population-based modeling for four conditions in heart failure human ventricular MID cells. **(A)** Population-based modeling of 1,000 variants for (Ai) HF, (Aii) HF + CO, (Aiii) HF + CO + RAN, and (Aiv) HF + CO + HW conditions. **(B)** EAD ratios under the four conditions.

Considering the potential influence of intercellular or intersubject variability on the reported findings, we built cell population models and performed additional simulations based on them. The simulation results are shown in Figure 13. It can be observed that EADs occurred occasionally under the HF condition, with a ratio of only 2.6%. Next, after considering the effects of CO, APDs of cell populations were generally prolonged, and the ratio of cells with EAD increased to 18.5%. The administration of ranolazine aggravated the situation, and the ratio of EAD cells increased dramatically to 58.2% (as shown in panel Aiii). In contrast, the addition of HW effectively alleviated the above arrhythmogenesis at the cellular level, which was evidenced by the complete suppression of EAD activities and the generally shortened APDs.

### 3.6 Simulating pseudo-ECGs based on a 2D realistic ventricular slice

**FIGURE 14:**
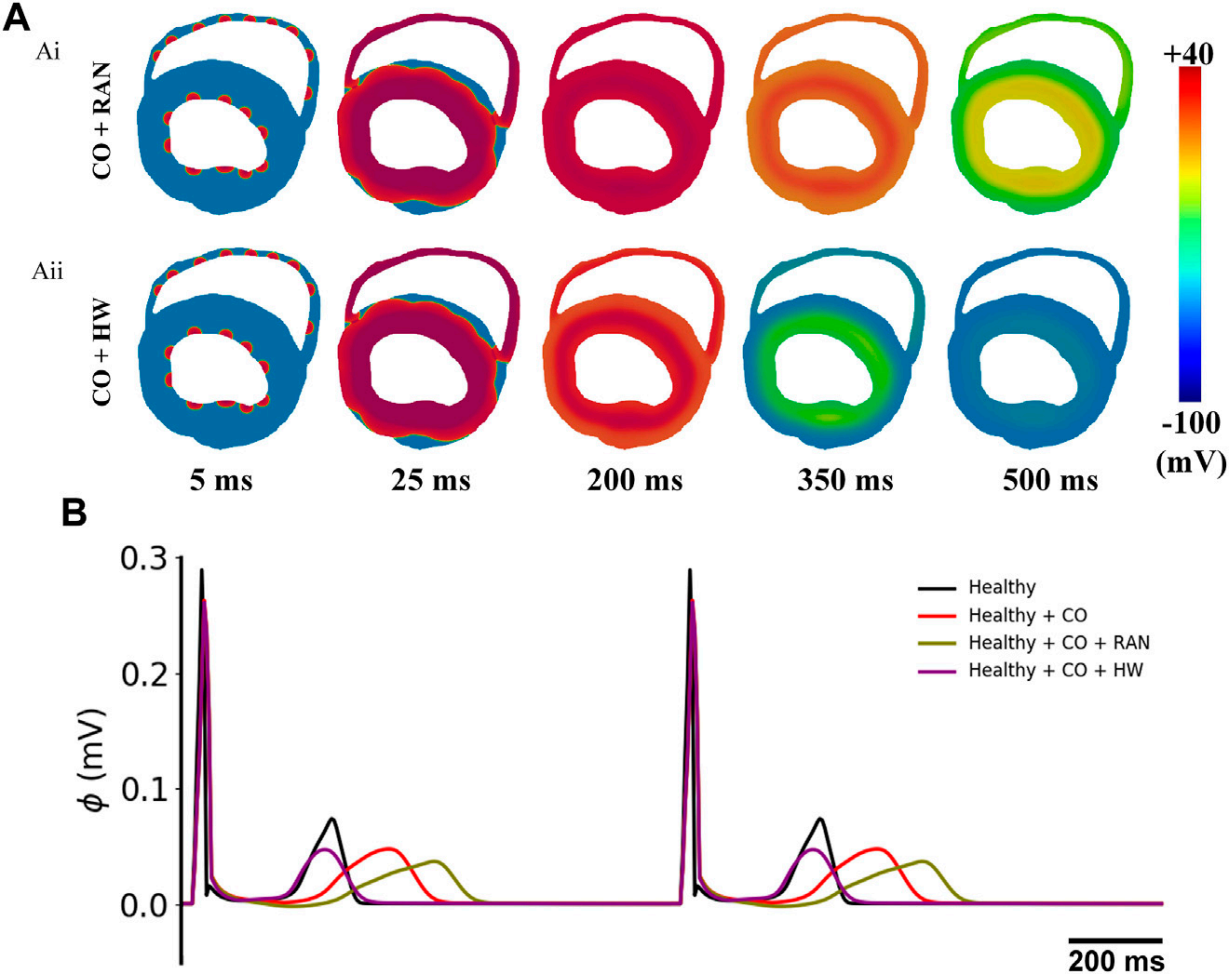
Simulation results of the influences of ranolazine and HW-0168 using a 2D realistic ventricular slice. **(A)** Propagation of excitation waves after applying ranolazine (Ai) or HW-0168 (Aii). **(B)** Pseudo-ECGs under different conditions.

To avoid the potential difference caused by the simplified model geometry, we conducted simulation experiments for two representative drugs, i.e., ranolazine and HW-0168, using a 2D realistic ventricular slice model. The simulation results are shown in Figure 14. It can be observed obviously that the tissue slice with ranolazine took more time to repolarize than that with HW-0168 (Figure 14A). In terms of the ECG, the 2D-based ECGs are consistent with the 1D-based ones (Figure 14B). For example, ranolazine further prolonged the QT interval based on CO and therefore exacerbated the proarrhythmic effect. On the other hand, HW-0168 still exerted the antiarrhythmic effects of ranolazine by restoring the QT interval.

## 4 Discussion

### 4.1 Main findings

The severe cardiotoxic consequences of CO urgently require an effective therapeutic strategy to treat them. In this study, we evaluated the efficacy of various multi-channel blockers and specific *I*_Kr_ activators against CO-induced ventricular arrhythmias in healthy and failing hearts. The major findings are as follows: 1) The tested existent antiarrhythmic drugs failed to rescue the heart from CO-induced arrhythmias, and most of them even aggravated the arrhythmogenic condition, which was evidenced by the more frequent EAD activities and decreased critical cell numbers for triggering ectopic beats. 2) In contrast, specific *I*_Kr_ activators demonstrated good efficacy according to the improved biomarkers at both cellular and tissue levels. All of the tested *I*_Kr_ activators restored the prolonged QT intervals in both healthy and heart failure conditions, and the EADs in MID cells were successfully suppressed as well. 3) In-depth case analysis with ranolazine and HW-0168 revealed the critical role of *I*_Kr_ in the CO-induced functional changes in cardiac electrophysiology, and neither *I*_CaL_ nor *I*_NaL_ blockers were able to offset the decreased repolarization forces caused by the CO-induced *I*_Kr_ inhibition. 4) Of note, the drug ranolazine was previously suggested as a potential strategy in dealing with CO-induced arrhythmogenesis due to its good efficacy demonstrated in rats, and the failure of ranolazine in the human tissue in this study hinted the crucial role of inter-species variances when determining the pharmacotherapeutic strategy.

### 4.2 Species-dependent effects of ranolazine for the treatment of CO-induced arrhythmias

Ranolazine was first suggested in Dallas et al.’s study (Dallas et al., 2012) for the treatment of CO-induced arrhythmias. Based on the experimental results obtained from rats, they proposed that CO-induced EADs arouse from the activation of NO synthase, which in turn leads to the NO-mediated nitrosylation of Na_V_1.5 and the enhanced *I*_NaL_. Correspondingly, the *I*_NaL_ inhibitor ranolazine abolished the EADs and was considered to be effective in dealing with CO-induced arrhythmias. Similarly, Morita et al. also observed the antiarrhythmic effects of ranolazine for its suppression of reentrant and multifocal ventricular fibrillation in rat ventricles (Morita et al., 2011). However, APs in rats are distinctly different from those in humans, and the such discrepancy may lead to species-dependent effects of the same drug. This hypothesis was explored in Al-Owais et al.’s study (Al-Owais et al., 2017), where the effects of ranolazine were examined in guinea pigs—a species with action potentials more closely resembling that of humans. Interestingly, ranolazine failed to abolish CO-induced EAD and even exacerbated such proarrhythmic factors.

Our simulations suggested that ranolazine exerted similar proarrhythmic effects in human hearts. Specifically, ranolazine further prolonged AP durations and QT intervals in healthy human simulations (Figure 4), while in heart failure conditions it led to more pronounced EADs in MID and EPI cells (Figure 6). The above model-dependent effects of ranolazine arose from the differences of *I*_Kr_, a major outward current responsible for the repolarization in human APs but are almost negligible in rat myocytes (Pandit et al., 2001). Although the *I*_NaL_ inhibition effects of ranolazine tend to suppress EAD, the drug can also reduce the repolarization force by inhibiting *I*_Kr_. Further assessment using a 1D homogenous ventricular strand consisting of only MID cells found that ranolazine decreased the critical cell number for triggering ectopic beats (from 68 to 58), which also suggested the increased arrhythmogenic risk of the drug. These findings provide new insights into the side effects of ranolazine on the treatment of CO-induced arrhythmias. They also highlighted that the drug effects obtained in rats need to be carefully interpreted in clinical trials due to the species-dependent differences.

### 4.3 *I*_Kr_ activator—A promising pharmacotherapy for the treatment of CO-induced arrhythmias

In addition to ranolazine, we evaluated more existent antiarrhythmic drugs to find potential drug strategies for CO-induced arrhythmias. Calcium current blockers were focused on in hopes of attenuating the depolarization force in the plateau phase and therefore shortening the action potential and the QT interval. However, none of the six drugs was able to rescue the heart from arrhythmogenesis, and most of them even worsened the conditions, evidenced by the further prolonged QT intervals and more frequently observed EAD activities. By analyzing the separate role of each channel current in the integral effect of multi-channel drugs, we found that blocking *I*_CaL_ and *I*_NaL_ was not able to offset the reduction of *I*_Kr_ by CO; furthermore, most of these multi-channel blockers also inhibited *I*_Kr_ with a relatively low affinity. Indeed, the hERG channel that conducts *I*_Kr_ is a highly sensitive target and it accounts for the majority of drug withdrawal events in the last 2 decades (Brown, 2004; Stockbridge et al., 2013; Villoutreix and Taboureau, 2015). On the other hand, there are few drugs available in the current antiarrhythmic category exerting *I*_Kr_ activating effects (Lei et al., 2018), making it difficult to find a proper drug strategy. We have also tried pinacidil (an *I*_KATP_ activator) in the model, but it did not produce any significant efficacy as well (data not shown). This can be attributed to the fact that the K-ATP channel barely opens under normoxic conditions due to its ATP-sensitive characteristic (Dart and Standen, 1995); therefore, the *I*_KATP_ would not make obvious differences even a high magnification ratio was used in the model.

In-depth analysis has demonstrated that *I*_Kr_ plays a major role in CO-induced arrhythmogenesis (Jiang et al., 2022). Considering that existent multi-channel antiarrhythmic drugs did not achieve idealized efficacy, we turned to evaluate the potential phrenological effect of specific *I*_Kr_ activators. In line with expectations, the simulation results showed that *I*_Kr_ activators could effectively reverse the proarrhythmic effects of CO. All the tested drugs notwithstanding in different doses restored AP and ECG morphologies almost to their control levels in healthy human simulations, and they also suppressed EADs and ectopic beats in heart failure human simulations. These findings suggest that the *I*_Kr_ activator is a promising pharmacotherapy for the treatment of CO-induced arrhythmias.

### 4.4 Potential limitations of this study

This study lacks validation of heart failure models. Though we have adopted a well-established cell model under heart failure conditions and replicated several known electrophysiological changes in failing hearts, for example, the prolonged APD (Akar and Rosenbaum, 2003; Lou et al., 2012), the decreased conduction velocity (Akar et al., 2004), the widened QRS complex (Shenkman et al., 2002; Sandhu and Bahler, 2004), and the prolonged QT interval (Davey et al., 2000; Medina-Ravell et al., 2003); however, we did not find enough tissue-level experimental data to validate other observations such as the flattened T-wave.

The above limitations shall not change the main conclusions of this study. Specifically, most of the observations and conclusions in the present study were based on the damaged cellular repolarization and the consequent QT prolongation in failing hearts, which were well-established in biological experiments (Davey et al., 2000; Medina-Ravell et al., 2003; Lou et al., 2012; Ng et al., 2014). In addition, for the EAD phenomenon, we adopted relatively conservative parameters (i.e., no EAD phenomenon occurred in pure heart failure conditions) to avoid exaggeration of the experimental results.

The experimental data on CO effects, drugs, and currents used in this study were obtained from different species, and the CO effects were obtained at room temperature. Interspecies differences and temperature dependence should be taken into account when interpreting and translating the results. The effects on APD in this study were measured in individual isolated ventricular myocytes, and the potential cell-coupling effects on the APD in high-dimensional models were not considered. Besides, the pathological model of CO was constructed based on experimental data obtained from different CORM-2 doses (10–30 μM) (Al-Owais et al., 2021), which should be considered in future studies. As for the drugs, the *I*_Kr_ activators proposed in this study for the treatment of CO-induced arrhythmias currently face some disadvantages and unknowns. Specifically, compared with the FDA-approved drugs such as ranolazine and amiodarone, *I*_Kr_ activators represented by HW-0168 are currently only used in biological experiments and simulation experiments, and their effective doses have not been clinically verified and side effects are not being disclosed. Moreover, whether these *I*_Kr_ activators interact with ion channels other than *I*_Kr_ remain unknown. If this is the case, then they must be treated as multiple-channel drugs and the potential offset or synergy effects among the involved ion currents should be considered.

Finally, according to our previous research review (Zhang et al., 2021), CO was also known to affect multiple cellular pathways other than the ion channels in this study. The present study mainly considered arrhythmias caused by changes in ionic currents directly induced by CO, without considering the mitochondrial toxicity of CO and some other complicated electrophysiological remodeling induced by cellular ischemia. Specifically, CO poisoning will increase ROS and RNS (Piantadosi, 2008), which further impair the chondrial energetics and can alter the intracellular calcium handling as well (Hegyi et al., 2021). This alteration will subsequently impact the expression and trafficking of channels (Sutanto et al., 2020). These cellular pathways warrant further investigations in the future.

## 5 Conclusion

In this study, we conducted an *in silico* assessment of the efficacy of some common antiarrhythmic drugs and specific *I*_Kr_ activators on CO-induced arrhythmias under healthy and heart failure conditions. We showed that existent antiarrhythmic drugs like ranolazine failed to exert therapeutic effects, and even worsened the arrhythmogenic situation in failing hearts. In contrast, specific *I*_Kr_ activators such as HW-0168 can effectively alleviate the proarrhythmic effects of CO, providing a promising pharmacotherapy for the treatment of CO-induced cardiotoxicity.

## Data Availability

The original contributions presented in the study are included in the article/Supplementary Materials, further inquiries can be directed to the corresponding authors.
