# Supplementary material for: In silico assessment of pharmacotherapy for carbon monoxide induced arrhythmias in healthy and failing human hearts
Source: Front Physiol. 2022 Nov 16;13:1018299. doi: 10.3389/fphys.2022.1018299 (PMC9709476; doi:10.3389/fphys.2022.1018299)
Supplement: Supplementary file 1 [file DataSheet1.DOCX]

Supplementary Material

# About this Supplementary Material

In this supplementary material, we present some fundamental methods and simulation results such as the construction of the baseline heart failure model and the effects of CO on healthy myocytes. Some of these contents might not new, as they are based on previous research; however, they are essential to the new findings presented in the paper. Therefore, we present these fundamental methods and results here, and more details can be obtained from the corresponding literatures cited at the end of this document.

# Supplementary Data

The source codes of this study could be downloaded via the link: <https://github.com/HuasenJiang/HuasenJiang-In-silico-assessment-of-pharmacotherapy-for-CO>

# Modeling Electrophysiological Changes in Failing Human Hearts

**Table S1.** Summary of the remodeled currents in cell models of heart failure.

| Ionic currents | Remodeling effects in heart failure | | |
| --- | --- | --- | --- |
|  | EPI* | MCELL* | ENDO* |
| *I*_Na_ | 74.1%↓ | 74.1%↓ | 74.1%↓ |
| *I*_NaL_ | 103.4%↑ | 103.4%↑ | 103.4%↑ |
| *I*_CaL_ | unchanged | unchanged | unchanged |
| *I*_to_ | 40.4%↓ | 37.6%↓ | 50.8%↓ |
| *I*_K1_ | 55.3%↓ | 52.7%↓ | 55.0%↓ |
| *I*_Kr_ | 45.9%↓ | unchanged | 27.3%↓ |
| *I*_Ks_ | 59.4%↓ | 49.5%↓ | 57.7%↓ |
| *I*_NaCa_ | 131.4%↑ | 90.0%↑ | 131.4%↑ |
| *I*_NaK_ | 40.2%↓ | 40.2%↓ | 40.2%↓ |
| SERCA | 42.3%↓ | 42.3%↓ | 42.3%↓ |

* ‘↑’ and ‘↓’ represent the augmentation and inhibition effects, respective.

## Heart failure-induced ion channel remodeling effects

The cell model of heart failure (HF cells) used in this study was mainly based on Elshrif et al.’s study (Elshrif et al., 2015), and the 1D tissue model of heart failure that consists of multiple HF cells was constructed accordingly. Specifically, Elshrif et al. (Elshrif et al., 2015) conducted a comprehensive *in silico* study on the heart failure-induced ion channel remodeling effects, as summarized in Table S1, and these effects were then incorporated into the ORd model.

## Simulated electrophysiological changes in HF conditions

The simulated cell and tissue-level electrophysiological changes in HF conditions are illustrated in Figure S1. Specifically, Figure S1A presents cellular action potentials of endocardial (ENDO), midcardial (MCELL/MID), and epicardial (EPI) in healthy and heart failure groups, which clearly shows that APDs of all three cell types were prolonged in heart failure. EPI cells achieved the largest increment (by 114.16%) in terms of APD_90_ among three cell types (73.48% and 66.67% for ENDO and MID cells, respectively).

The changes of excitation conduction and electrocardiogram were investigated using the 1D transmural tissue model, as depicted in Figure 1B. Consistent to the single cell results, the excitation wavelength became significantly wider in heart failure tissue (Figure S1Bii) compared to the healthy condition (Figure S1Bi). In addition, the transmural gradient was diminished due to the larger increment of APD in EPI and ENDO cells than MID cells. The decreased transmural gradient was also reflected in pseudo-ECGs (Figure S1Biii), where the upward T-wave was replaced by that with a flattened and slightly inversed morphology. Besides, a moderate QRS complex widening could be observed (Shenkman et al., 2002; Sandhu and Bahler, 2004). This was attributed to the slowed conduction that arouse from the reduced *I*_Na_.


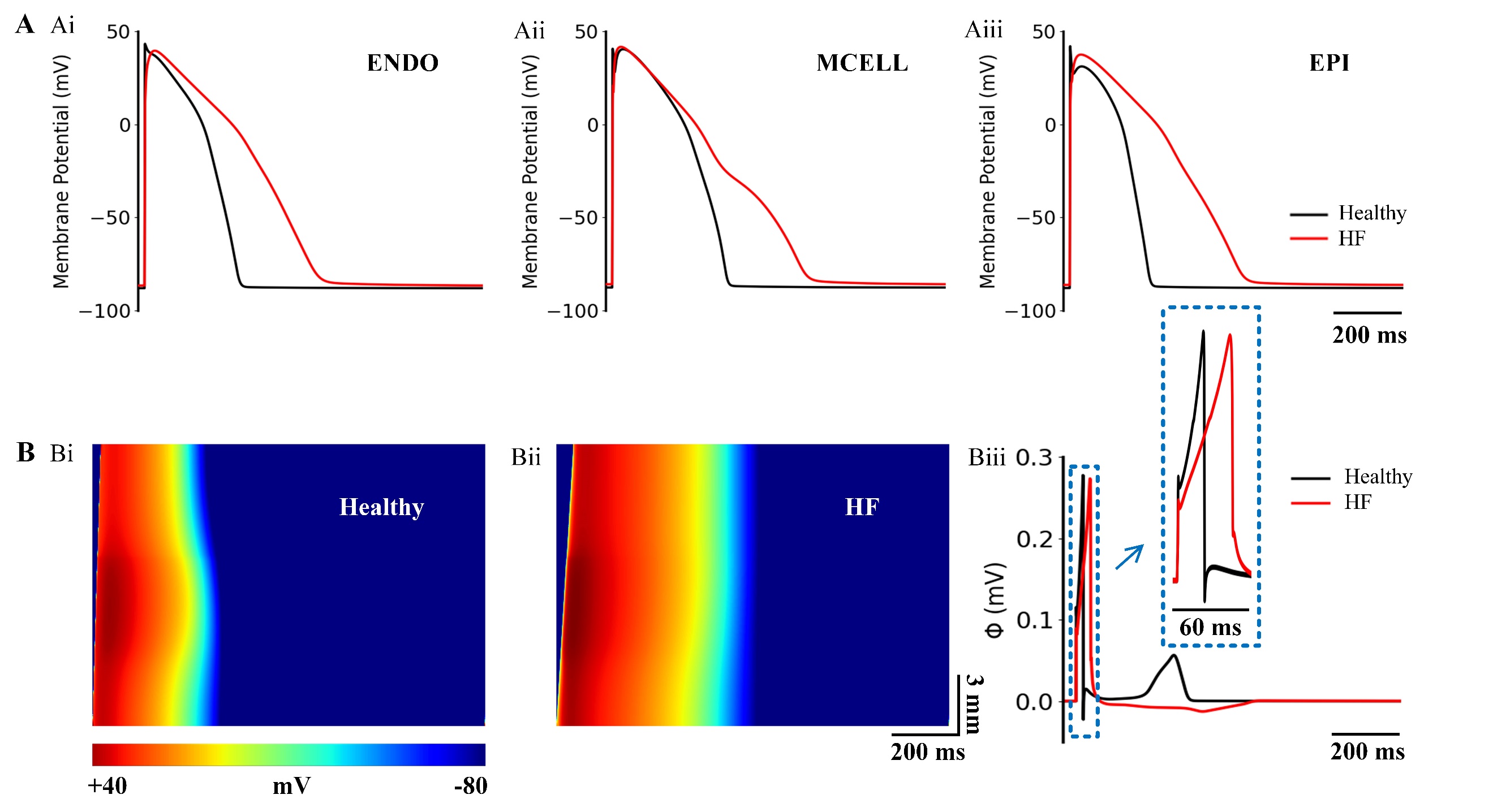


**Figure S1.** Cell and tissue-level electrophysiological changes in heart failure. **(A)** Comparisons of action potentials of three cell types in healthy and heart failure conditions. **(B)** Simulation results of 1D ventricular tissue model: (Bi-Bii) Space-time plots of action potential propagations in healthy and heart failure groups; (Biii) The comparison of pseudo-ECGs.

# Modeling Effects of CO on Cardiac Electrophysiology

## Effects of CO on ion channels

For the CO condition, we modeled its effects by applying scaling factors to the corresponding ion channel conductance as in Al-Owais et al. (Jiang et al., 2022)(Table 2), which were obtained from experimental studies (Scragg et al., 2008; Dallas et al., 2012; Elies et al., 2014; Liang et al., 2014; Al-Owais et al., 2017). In this study, we adopted the effects of CORM-2 (i.e., a CO-releasing molecule) to represent the CO condition to be consistent with experiments.

**Table S2.** Changes in ionic currents by CO.

| **Ionic currents** | | **CO/CORM-2 effect*** | | **Ref** |
| --- | --- | --- | --- | --- |
| *I*_Na_ | 53%↓ | | Dallas et al. (2012) | |
| *I*_NaL_ | 105%↑ | | Dallas et al. (2012) | |
| *I*_CaL_ | 53%↓ | | Scragg et al. (2008) | |
| *I*_Kr_ | 44%↓ | | Al-Owais et al. (2017) | |
| *I*_K1_ | 34%↓ | | Liang et al. (2014) | |

* ‘↑’ and ‘↓’ in this column represent augmentation or inhibition.Simulated electrophysiological changes in HF conditions

## Effects of carbon monoxide on healthy myocardial myocytes and tissues

In our previous study, we have comprehensively simulated the influences of carbon monoxide on ventricular tissues and revealed the associated arrhythmogenic mechanisms (refer to (Jiang et al., 2022) for more details). For the integrity of this study, some of the main results are briefly introduced here.

Figure S2 presents the simulated effects of CO in healthy conditions. In the cellular level, CO prolonged APDs of all the three cell types, with APD_90_ of ENDO, MID, and EPI cells being increased by 43.18%, 36.11%, and 50.44%, respectively. The prolonged AP further led to conduction abnormalities of excitation waves in the tissue level. Figure S2B show simulation results of the propagation of excitation waves in a 1D heterogeneous tissue strand model under healthy (Figure S2Bi) and CO conditions (Figure S2Bii). It can be clearly observed that the excitation wavelength became wider under the CO condition compared to the healthy condition, which is similar to that in heart failure. The conduction velocities of excitation waves were 0.70 m/s and 0.54 m/s under healthy and CO conditions. In terms of the ECG, as in Figure S2Biii, CO caused a significant QT interval prolongation. An increase in T_peak_-T_end_ (Tpe) was also observed, suggesting an increased susceptibility to unidirectional conductions and reentry arrhythmias.


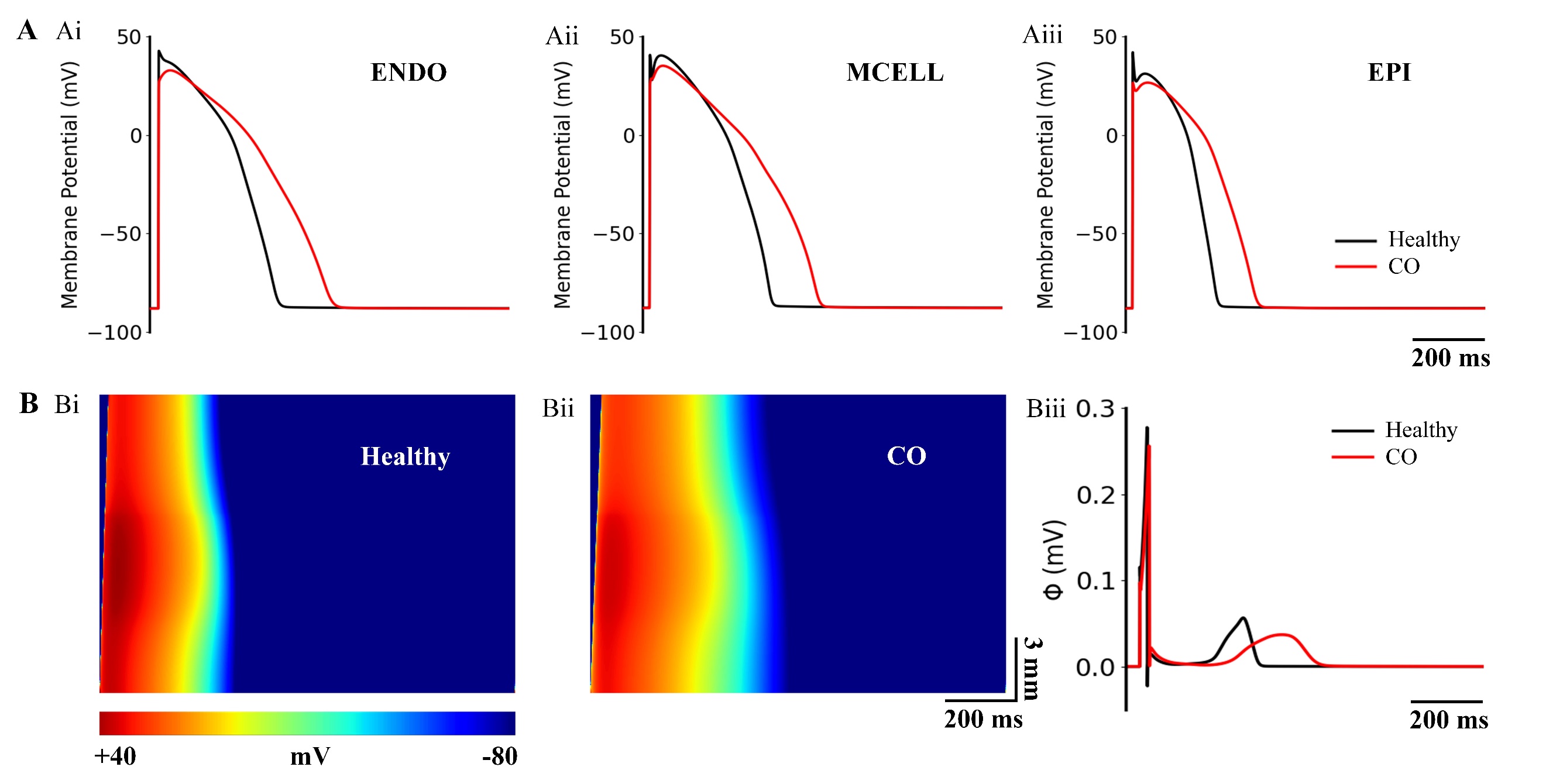


**Figure S2**. Simulated effects of CO in cell and tissue models. **(A)** Comparisons of action potentials of three cell types in healthy and CO conditions. **(B)** Simulation results of 1D ventricular tissue model: (Bi-Bii) Space-time plots of AP propagations in healthy and CO groups; (Biii) The comparison of pseudo-ECGs.

# References

Al-Owais, M. M., Hettiarachchi, N. T., Kirton, H. M., Hardy, M. E., Boyle, J. P., Scragg, J. L., et al. (2017). A key role for peroxynitrite-mediated inhibition of cardiac ERG (Kv11.1) K+ channels in carbon monoxide-induced proarrhythmic early afterdepolarizations. *FASEB J.* 31, 4845–4854. doi: 10.1096/fj.201700259R.

Dallas, M. L., Yang, Z., Boyle, J. P., Boycott, H. E., Scragg, J. L., Milligan, C. J., et al. (2012). Carbon monoxide induces cardiac arrhythmia via induction of the late Na+ current. *Am. J. Respir. Crit. Care Med.* 186, 648–656. doi: 10.1164/rccm.201204-0688OC.

Elies, J., Dallas, M. L., Boyle, J. P., Scragg, J. L., Duke, A., Steele, D. S., et al. (2014). Inhibition of the cardiac Na+ channel Nav1.5 by carbon monoxide. *J. Biol. Chem.* 289, 16421–16429. doi: 10.1074/jbc.M114.569996.

Elshrif, M. M., Shi, P., and Cherry, E. M. (2015). Representing Variability and Transmural Differences in a Model of Human Heart Failure. *IEEE J. Biomed. Heal. Informatics* 19, 1308–1320. doi: 10.1109/JBHI.2015.2442833.

Jiang, H., Zhang, S., Bi, X., Ma, W., and Wei, Z. (2022). Proarrhythmic effects of carbon monoxide in human ventricular tissue: insights from computational modeling. *Comput. Biol. Med.* 140. doi: 10.1016/j.compbiomed.2021.105066.

Liang, S., Wang, Q., Zhang, W., Zhang, H., Tan, S., Ahmed, A., et al. (2014). Carbon monoxide inhibits inward rectifier potassium channels in cardiomyocytes. *Nat. Commun.* 5. doi: 10.1038/ncomms5676.

Sandhu, R., and Bahler, R. C. (2004). Prevalence of QRS prolongation in a community hospital cohort of patients with heart failure and its relation to left ventricular systolic dysfunction. *Am. J. Cardiol.* 93, 244–246. doi: 10.1016/j.amjcard.2003.09.053.

Scragg, J. L., Dallas, M. L., Wilkinson, J. A., Varadi, G., and Peers, C. (2008). Carbon monoxide inhibits L-type Ca2+ channels via redox modulation of key cysteine residues by mitochondrial reactive oxygen species. *J. Biol. Chem.* 283, 24412–24419. doi: 10.1074/jbc.M803037200.

Shenkman, H. J., Pampati, V., Khandelwal, A. K., McKinnon, J., Nori, D., Kaatz, S., et al. (2002). Congestive heart failure and QRS duration: Establishing prognosis study. *Chest* 122, 528–534. doi: 10.1378/chest.122.2.528.
